# Current Landscape of Breast Cancer Imaging and Potential Quantitative Imaging Markers of Response in ER-Positive Breast Cancers Treated with Neoadjuvant Therapy

**DOI:** 10.3390/cancers12061511

**Published:** 2020-06-09

**Authors:** Ella F. Jones, Deep K. Hathi, Rita Freimanis, Rita A. Mukhtar, A. Jo Chien, Laura J. Esserman, Laura J. van’t Veer, Bonnie N. Joe, Nola M. Hylton

**Affiliations:** 1 Department of Radiology and Biomedical Imaging, University of California, San Francisco, CA 94115, USA; Deep.Hathi@ucsf.edu (D.K.H.); Rita.Freimanis@ucsf.edu (R.F.); Bonnie.Joe@ucsf.edu (B.N.J.); Nola.Hylton@ucsf.edu (N.M.H.); 2 Department of Surgery, University of California, San Francisco, CA 94115, USA; Rita.Mukhtar@ucsf.edu; 3 School of Medicine, Helen Diller Family Comprehensive Cancer Center, University of California, San Francisco, CA 94115, USA; Jo.Chien@ucsf.edu (A.J.C.); Laura.Vantveer@ucsf.edu (L.J.v.V.); 4 Department of Surgery, Helen Diller Family Comprehensive Cancer Center, University of California, San Francisco, CA 94115, USA; Laura.Esserman@ucsf.edu

**Keywords:** breast cancer, estrogen receptor (ER), neoadjuvant therapy, mammography, MRI, PET, quantitative imaging, ultrasound, X-ray

## Abstract

In recent years, neoadjuvant treatment trials have shown that breast cancer subtypes identified on the basis of genomic and/or molecular signatures exhibit different response rates and recurrence outcomes, with the implication that subtype-specific treatment approaches are needed. Estrogen receptor-positive (ER+) breast cancers present a unique set of challenges for determining optimal neoadjuvant treatment approaches. There is increased recognition that not all ER+ breast cancers benefit from chemotherapy, and that there may be a subset of ER+ breast cancers that can be treated effectively using endocrine therapies alone. With this uncertainty, there is a need to improve the assessment and to optimize the treatment of ER+ breast cancers. While pathology-based markers offer a snapshot of tumor response to neoadjuvant therapy, non-invasive imaging of the ER disease in response to treatment would provide broader insights into tumor heterogeneity, ER biology, and the timing of surrogate endpoint measurements. In this review, we provide an overview of the current landscape of breast imaging in neoadjuvant studies and highlight the technological advances in each imaging modality. We then further examine some potential imaging markers for neoadjuvant treatment response in ER+ breast cancers.

## 1. Introduction

Gene expression profiling has given us a better understanding of breast cancer as a heterogeneous disease and led to the identification of distinct molecular subtypes [1,2]. The major intrinsic subtypes, classified as luminal A, luminal B, basal-like, and human epidermal growth factor receptor 2 (HER2)-enriched, reflect the heterogeneity within breast cancer, with each subtype having a unique biology, prognosis, and response to treatment [3,4,5,6,7]. Luminal breast cancers comprise >70% of all breast cancers [8]. A large majority of luminal breast cancers are estrogen receptor-positive (ER+). While patients with ER+ tumors have lower recurrence rates within the first five years compared to other subtypes, their risk persists over decades, with >50% of recurrences occurring after five years, and are responsible for the majority of breast-cancer related deaths [9,10]. While endocrine treatments using tamoxifen and aromatase inhibitors (AIs) are generally found to be effective in ER+ breast cancers, recurrent tumors are often resistant or become resistant to these treatments. Therefore, there is a clear unmet clinical need to develop new tools to improve the assessment and to optimize the treatment of ER+ breast cancers.

Clinical studies [11,12] have shown similar results in disease-free and overall survival in breast cancer patients treated with neoadjuvant chemotherapy (NAC) vs. those with adjuvant chemotherapy. These findings have shifted breast cancer management to greater use of NAC in patients with high likelihood of response to chemotherapy. NAC has several clinical benefits. It enables tumor downstaging for breast conservation surgery, provides in vivo information about the primary tumor to inform therapeutic efficacy, and importantly, it provides predictive information about the benefit of adjuvant therapy [13]. Pathologic complete response (pCR) to NAC (eradication of invasive disease in the breast and lymph nodes after NAC) also serves as a surrogate endpoint, providing important prognostic information [14,15,16] for treatment intervention and accelerating new drug development.

Recently, neoadjuvant endocrine therapy (NET) trials have emerged as platforms to identify new therapeutic approaches for ER+ breast cancers [17,18,19,20]. While pCR has been adopted as a short-term endpoint in NAC trials for its use in accelerated drug approval [21], its effectiveness as a surrogate of long-term outcome in NET trials is less compelling [22]. The main challenge is that ER+ cancers have a low rate of pCR, and non-pCR does not infer poor outcomes. In fact, many ER+ patients failing to achieve short-term pCR achieve excellent long-term outcomes with adjuvant endocrine therapy [19].

Residual cancer burden (RCB) index [23] is another pathological measure based on tumor size, tumor cellularity, and nodal status. It is increasingly being used to measure NAC response [24,25,26,27]. However, the adoption of RCB in NET studies suffers from similar challenges as in pCR. In the NeoPAL study [28], RCB was the primary endpoint to assess the efficacy of combining letrozole with palbociclib (LETPAL) vs. standard chemotherapy in 106 ER+ breast cancer patients. Of the 52 patients in the LETPAL arm, only 4 (7.7%) achieved RCB 0/I after 28 daily regimens. Although the NeoPAL trial did not meet the accrual goal, the slow response and low RCB 0/I rate were demonstrated in this study.

The proliferation antigen Ki-67 is highly expressed in all cell cycles phases except for G_0_ [29]. It has been proposed as a measure to assess the potential efficacy of endocrine treatments in NET trials [10]. The collective evidence from the P024 [30], IMPACT [31], and ACOSOG Z1031 (Alliance) [32] trials showed that treatment-induced reduction in Ki-67 level in post-menopausal ER+ breast cancer patients is associated with disease-free (DFS) and relapse-free survival (RFS). However, some tumors present a paradoxical increase in Ki-67 value from baseline despite the initial on-treatment reduction [33]. Other caveats include analytical consistency, intra- and inter-reader reproducibility, and scoring approaches. An international Ki-67 in Breast Cancer Working Group was formed to address some of these problems. Analyses of 30 cases of ER+ breast cancer performed by 14 laboratories from 6 countries using 7 different scanners and 10 software were reported in 2019 [34]. By adopting an automated machine-based scoring method, the intraclass correlation coefficient for automated average Ki-67 was 0.83 and for maximum Ki-67 was 0.63. Results from this group indicated that the maximum Ki-67 score is suboptimal for consistent measurement of proliferation. While the automated average Ki-67 scoring shows promise in reproducibility, optimal timing for when to obtain the on-treatment Ki-67 value still needs to be determined. Importantly, the use of Ki-67 also needs to be demonstrated in premenopausal patients for future clinical validations.

A multivariate analysis of Ki-67, tumor size, nodal involvement, and ER status in the P024 study had led to the development of the preoperative endocrine prognostic index (PEPI) scoring system [35]. The PEPI score weighs these independent predictors, based on their hazard ratio of relapse risk. The end result is that a PEPI score of 0 at surgery translates to a low relapse risk, and further adjuvant chemotherapy would be unnecessary. A PEPI score of 4+, on the other hand, indicates a higher risk of early relapse that warrants triaging patients to adjuvant chemotherapy. While the PEPI scoring system shows promise in predicting high and low relapse risks, further study is needed to clearly define the “cut-off” of intermediate relapse risk and how to manage patients with intermediate scores of PEPI 1–3. Other limitations include: (1) sampling error due to intra-tumoral heterogeneity; (2) timing of the “on-treatment” Ki-67 measurement and (3) usefulness of Ki-67 in treatment-induced apoptosis [19].

Collectively, the aforementioned pathological-based surrogate endpoints, obtained at surgery, offer a snapshot of tumor response to neoadjuvant treatment. To gain broader insights into tumor heterogeneity, ER biology, and the timing of surrogate endpoint measurement, non-invasive monitoring of the disease in response to treatment would be extremely valuable. Indeed, imaging has been an integral part of neoadjuvant studies [36]. Mammography, ultrasound, magnetic resonance imaging (MRI), and positron emission tomography combined with computed tomography (PET-CT) are all essential to the detection, staging, and monitoring of breast cancer. In this review, we summarize the current landscape of breast imaging in neoadjuvant studies and highlight the technological advances in each modality. We then further examine some potential imaging markers for neoadjuvant treatment response in ER+ breast cancers.

## 2. Imaging of Neoadjuvant Treatment Response in Breast Cancers

In the NAC setting, quantitative imaging plays a vital role in assessing the response of the intact primary tumor to targeted systemic therapies. Treatment-induced change in the primary tumor may serve as a surrogate marker for the effect of therapy, providing valuable information on drug efficacy for subsequent clinical decision making. In the NET setting, however, there is no consensus on the best imaging method to use for assessing response. Most of the NET studies are limited to tumor size measurements based on clinical exam, mammogram or ultrasound [37]. A review of more advanced quantitative imaging tools used in the NAC setting may provide insights into potentially more accurate markers for ER+ tumor response to NET.

### 2.1. Mammography

Screening mammography (MG) is a low-energy (26–34 kVp) X-ray imaging technique that is used in asymptomatic women for detecting microcalcifications, masses, architectural distortion and asymmetries that may reflect breast cancer. Diagnostic mammography is often used to assess findings from screening mammography, and in patients with symptoms or signs such as palpable mass, skin thickening or nipple discharge. Evaluation is enhanced by noting change in appearance compared to previous mammograms. Mammography together with breast ultrasound (US) has been used to measure residual tumor burden during neoadjuvant therapy. While the resolution of some baseline mammographic features such as microcalcifications and spiculation of masses are associated with response [38], the use of mammography may be of limited value during neoadjuvant therapy due to variable accuracy and sensitivity to changes in residual tumor burden [39].

Several studies have shown that the extent of tumor size reduction by mammography correlates poorly to pathologic tumor size and pCR. In a study of 130 patients receiving NAC, there was a stronger correlation between pathologic tumor size and MRI tumor volume (*r* = 0.55) than that compared to mammographic extent of microcalcifications (*r* = −0.12) [40]. Interestingly, in the ER- population (*N* = 67), MRI and mammography performed similarly (MRI, *r* = 0.39 vs. MG, *r* = 0.36, *p* = 0.84), while MRI enhancement had higher correlation to pathologic tumor size than mammography in ER+ patients (*N* = 67; MRI, *r* = 0.55 vs. MG, *r* = 0.19, *p* = 0.02). Other studies also found no statistically significant correlation between the measure of calcifications extent and pCR (*p* = 0.06) or MRI enhancement (*p* = 0.12), although the presence of microcalcifications at post-treatment was used to identify candidates for breast-conserving surgery [41].

Given the very high resolution of mammography, microcalcifications are best seen with this imaging technique. Calcifications may be driven by the presence of necrotic tissue within a malignant mass or may reflect necrosis or other processes within ductal carcinoma in situ (DCIS). While an increase in malignant-appearing calcifications associated with known DCIS may signify disease progression, studies have shown that residual mammographic microcalcifications following treatment may not always be associated with active malignant disease [42,43]. With this uncertainty, the handling of residual post-treatment mammographic microcalcifications remains controversial. Moreover, the likelihood of malignancy in residual calcifications may be subtype-specific. In particular, malignant residual calcifications are more likely found in ER+ patients [42] and are linked to poor response in luminal disease with DCIS [44,45]. Therefore, breast cancer subtype must be considered when mammography is used to monitor response to neoadjuvant therapy.

**Figure 1:**
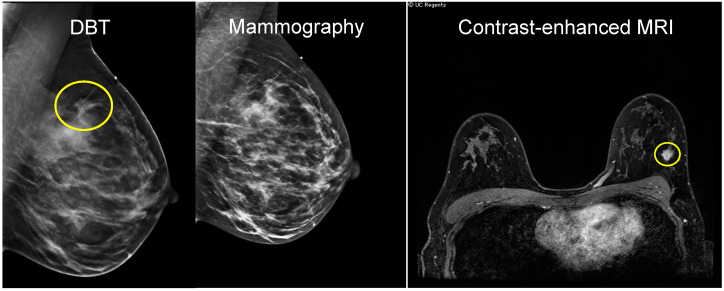
An invasive ductal carcinoma (IDC) presenting as palpable lump, best seen on tomosynthesis slices of DBT (**left**), with asymmetry and architectural distortion. The heterogeneously dense breast tissue obscures the finding on the 2D mammographic image (**center**). Ultrasound (not shown) demonstrated a hypoechoic lesion and was redemonstrated by contrast-enhanced MRI with an irregular mass measured at 1.2 × 1.1 × 1.4 cm (**right**).

For many years, mammography has been performed utilizing X-ray beam projections resulting in 2D images, which are subject to potential tumor masking by dense breast tissue. Digital breast tomosynthesis (DBT) is a newer type of mammography that collects image data at multiple projection angles while the breast is compressed in standard planes. The resulting reconstructed image is a 3D compressed breast volume [46]. The volume may also be viewed as a series of individual slices, which enhances lesion boundaries and retains morphological information of masses, thus improving visualization in dense tissue and differentiation between benign and malignant processes [47]. Compared to 2D mammography, DBT showed a more accurate estimation of tumor size, especially those with diameters < 2 cm and in dense breasts [48] (Figure 1). In a study of 51 patients undergoing NAC, tumor size and prediction of pCR were compared among 2D mammography (MG), DBT, ultrasound (US), and MRI. While MRI-measured tumor size had the highest correlation with pathologic tumor size (intraclass correlation coefficients (ICC): MRI = 0.83; MG = 0.56; DBT = 0.63 and US = 0.55), both MRI and DBT were predictive of pCR, more so than mammography and US (area under the receiver operating characteristic curve (AUC): MRI = 0.92; DBT = 0.84; MG = 0.72; US = 0.75) [49,50].

**Figure 2:**
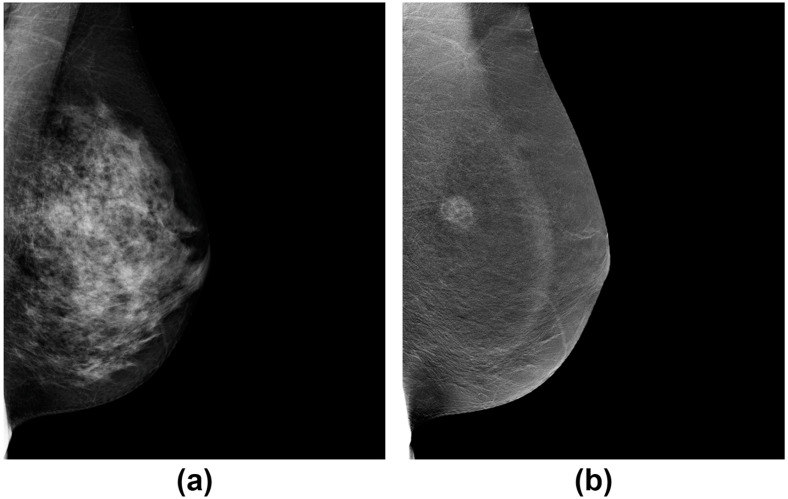
An example of a mammographically occult mass in a dense breast (**a**) on the low-energy image but is demonstrated on the dual-energy recombined contrast-enhanced image (**b**) (CESM).

In recent years, the development and clinical implementation of contrast-enhanced spectral mammography (CESM) have shown considerable promise in therapy monitoring. CESM combines iodine-based intravenous contrast agents with dual-energy (low at 24–35 kVp and high 40–47 kVp) [51] image acquisition to capture morphologic and microvessel density (MVD) features of the breast [52]. The resulting dual-energy recombined image has high sensitivity for detecting lesions in dense breasts that are otherwise occult on conventional mammography [53] (Figure 2) [54]. More recent studies of CESM in monitoring breast cancer treatment response have shown that CESM measured tumor size at pre- and during treatment time points is highly correlated to MRI [55,56] with comparable sensitivity and specificity to predict treatment response. However, CESM is not without limitations. Given the need of iodine-based contrast agents for the exam, there is a risk of adverse reactions in some patients. False positives in dealing with fibroadenomas, hamartomas, intra-mammary nodes, and fat necrosis have significantly reduced the specificity of this method as a primary technique for breast cancer. Since CESM and DBT are promising mammographic techniques that may potentially add values to treatment monitoring, studies in large patient populations will be important to validate the initial findings and to stratify imaging phenotypes by subtype.

### 2.2. Ultrasound

Compared to mammography, ultrasound-measured breast tumor size showed higher correlation to pathologic tumor size (*r* = 0.68 (US) vs. *r* = 0.44 (MG)) [57]. In a retrospective cohort of 196 patients, US was able to estimate post-treatment residual tumor size more accurately than mammography (59.6% (US) vs. 31.7% (MG), *p* < 0.001) [58]. In the prediction of pCR, however, there was no statistically significant difference in specificity and sensitivity between the two modalities with the AUC of 0.741 (US) vs. 0.784 (MG).

Interestingly, studies have shown similar subtype-driven effects in ultrasound as in mammography. In a study of 159 patients, the percent change in US-measured tumor size at mid-treatment was linearly associated with the RCB index score in triple-negative (TN) and hormonal positive (HR+; ER or progesterone (PR) receptor positive or both) subtypes, but not in HER2+ tumors [59]. US imaging features such as the absence of posterior acoustic shadowing and spiculation were significant predictors of pCR in HER2+ tumors [38]. On the other hand, no feature was found to associate with response in the TN subtype, and the presence of posterior shadowing was associated with HR+/HER2− breast cancers [60]. However, axillary lymph node characterization, which is most accurately managed by ultrasound [36], is largely independent of subtype. While there remains an association between RCB and the number of axillary lymph nodes with residual disease, there is no significant effect (*p* > 0.05) of subtype on this association [59]. Indeed, the sensitivity, specificity, and positive predictive value (PPV) for lymph node detection were found to be independent of subtype, although the negative predictive value (NPV) was highest for TN and lowest for HER2+ cancers [61].

As with mammography, there has been a recent push in the development and deployment of contrast-enhanced ultrasound (CEUS) for imaging lesion vascularity. A meta-analysis of nine studies involving 424 patients [62,63] showed that CEUS had pooled sensitivity of 87% and specificity of 84%, which is comparable to dynamic contrast-enhanced (DCE) MRI and PET using [^18^F]fluorodeoxyglucose ([^18^F]FDG) [64]. However, publication bias, pooled data, and small sample sizes of comparative studies may overestimate the diagnostic accuracy of CEUS. Nonetheless, CEUS may hold promise as an emerging imaging technique, and further investigations of its accuracy by subtype would be of interest.

Tissue remodeling from cell migration to cell alignment and collagen crosslinking are steps that lead to stiffening of the extracellular matrix (ECM) in tumorigenesis [65]. Shear-wave elastography (SWE) estimates tissue stiffness via a mechanical pulse generated by the ultrasound transducer [66]. Pre- and post-treatment stiffness has been correlated to RCB and residual tumor burden, independent of subtype [67,68]. However, more studies are required for establishing SWE in clinical care.

### 2.3. Magnetic Resonance Imaging

MRI is considered an accurate method to assess treatment response in breast cancer [69]. Contrast-enhanced MR imaging (CE-MRI) is the most common MRI technique for functional assessment of breast tissue. It involves serial image acquisition at time points before and after the administration of gadolinium contrast agent. CE-MRI derived tumor volume [70,71], tumor size [72,73], and pharmacokinetic (PK) parameters [74,75,76,77], including volume transfer constant (*K^trans^*), exchange rate constant (*k*_ep_), extravascular extracellular space volume (*v_e_*), and early contrast uptake, are increasingly used to assess response to NAC.

#### 2.3.1. Tumor Volume

**Figure 3:**
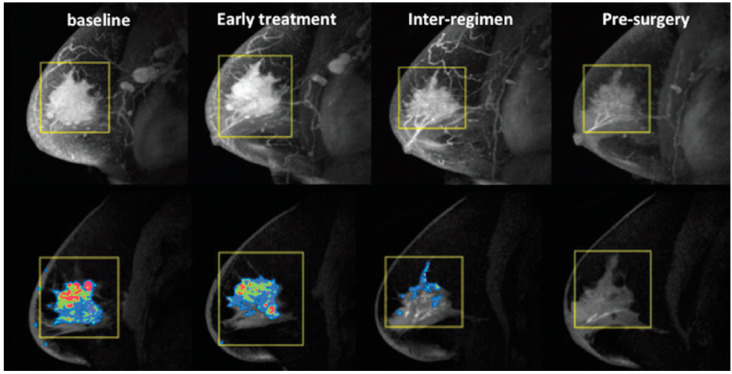
An example of using MRI functional tumor volume (FTV) to monitor a breast cancer patient during neoadjuvant chemotherapy. Shown here are maximum intensity projection images (top row) and corresponding FTV maps (bottom row) for a patient with an excellent clinical response. From left to right, FTV measurements were 48.5, 35.4, 5.6, and 0 cm^3^ at baseline, early treatment, inter-regimen, and pre-surgery time points, respectively.

When examining the accuracy of CE-MRI quantitative measurements for prediction of pCR to NAC in breast cancer, tumor volume showed the highest sensitivity/specificity compared to tumor size and PK parameters [78]. Indeed, functional tumor volume (FTV) [79], computed by summing all voxels above the percent enhancement (PE) and signal-enhancement-ratio (SER) thresholds, was predictive of response during NAC in the American College of Radiology Imaging Network (ACRIN) 6657/I-SPY 1 TRIAL [80,81] (Figure 3). In this study of 216 patients, the pre-/post-treatment FTV ratio was more predictive of pCR early in the treatment (AUC = 0.70) than the longest diameter (AUC = 0.64), SER (AUC = 0.57), and clinical exam (AUC = 0.56). Reduction in FTV reflects the angiogenic changes that may precede size reduction, demonstrating a clear advantage as a marker for tumor response to guide earlier treatment invention for better clinical outcomes.

Prediction of pCR by FTV can be further improved by adjusting the PE and SER thresholds according to the breast cancer subtype [82]. In a subset of 116 patients from the ACRIN 6657/I-SPY 1 TRIAL [83], the estimated AUC in the HR+/HER2− group at mid-treatment was improved from 0.70 to 0.90 when the PE threshold was increased from 70% to 130%. Likewise, the pCR prediction in the HER2+ and TN groups also improved when the PE/SER thresholds were adjusted upward. These findings highlight the heterogeneity of breast cancer, requiring not only personalized treatment, but also optimized subtype-specific imaging parameters to accurately assess treatment response.

#### 2.3.2. Pharmacokinetic Parameters

In addition to tumor volume, PK parameters may reveal tumor vascular response to therapeutic effects [84]. From a collection of studies with small cohorts [76,84,85,86,87,88,89,90,91], reduction in *K^trans^* and *k_ep_* values after NAC is generally found in responders, whereas non-responders showed minimal reduction, or, in some cases, an increase in *K^trans^* and *k_ep_*. In a more recent meta-analysis of 14 quantitative MRI studies of breast tumor response to NAC [92], three studies [85,86,89] with PK measurements were extracted with the combined sensitivity and specificity at 84.1% and 81.3%, respectively. These initial results suggest that *K^trans^* and *k_ep_* representing perfusion and vascular permeability may be important parameters in identifying responders from non-responders. While the PK parameters are investigational and the sample size of all these studies are too small for subtype analyses, the initial findings are nonetheless promising for further investigations.

#### 2.3.3. Background Parenchymal Enhancement

Contrast enhancement in the breast parenchyma, termed background parenchymal enhancement (BPE), is a phenomenon observed on breast MRI, in which normal breast tissue shows enhancement in various patterns from the uptake of contrast agent. The BPE level is influenced by endogenous sex hormones and varies throughout the menstrual cycle in premenopausal women [93,94,95]. While the exact biological mechanism of BPE is unknown, histopathologic exams of the contralateral fibroglandular tissues from prophylactic mastectomy in 80 premenopausal women found a strong correlation between BPE and microvessel density (MVD), endothelial density (CD34), glandular concentration, and vascular endothelial growth factor (VEGF) [96]. This study, though preliminary, suggests that women with high BPE may have higher epithelial density and greater glandular structure that may contribute to breast cancer risk [97].

**Figure 4:**
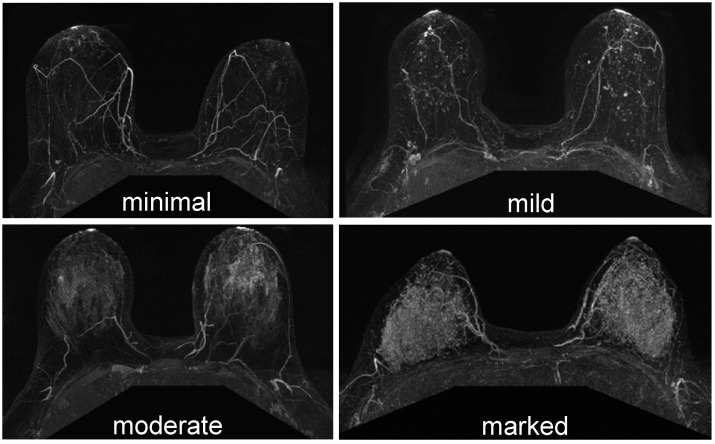
Examples from the Breast Imaging-Reporting and Data System (BI-RADS) lexicon for contrast-enhanced MRI, showing the different degrees of background parenchymal enhancement (BPE) in the breast: minimum, mild, moderate, and marked enhancement.

In current clinical practice, BPE is determined by qualitative visual assessment and is reported as minimal, mild, moderate, or marked, as seen on early post-contrast images in accordance with the American College of Radiology’s Breast Imaging Reporting and Data System (BI-RADS) [98,99] (Figure 4). Quantitative approaches based on percent enhancement (PE) within the region or volume of interest in 2D or 3D have also been used to describe BPE. A recent review of BPE on breast MRI by Liao et al. has summarized the variation of imaging protocol and the different approaches of BPE quantification [100]. Without the consensus of optimal timing for measurements and no standardization of BPE calculations, it is difficult to implement quantitative BPE to clinical practice.

Since the initial report linking BPE to the risk of breast cancer diagnosis [101], many subsequent studies have demonstrated that treatment-induced changes of BPE in the ipsilateral or contralateral breast may also serve as a predictor of tumor response [100]. In the NAC setting, post-treatment reduction of BPE from baseline was associated with tumor response or pCR [102,103,104,105]. However, for ER+/HER2− breast cancer patients undergoing endocrine therapy, an increase in post-treatment BPE was associated with low PEPI score [106], and those with low baseline BPE (minimal/mild) were found to have better response [107]. These initial results underscore the impact of systemic therapy on the breast tissue vascularity and the influence of exogenous ER modulators on the BPE level in response to treatment. The collective evidence so far points to the potential use of BPE as a biomarker for risk and response assessment for breast cancers, including the ER+/HER2− subtype.

#### 2.3.4. Apparent Diffusion Coefficient

Diffusion-weighted imaging (DWI) is another promising MRI technique for assessing tumor functionality in breast cancer. DWI measures the thermal motion of water molecules, resulting in an estimation of the apparent diffusion coefficient (ADC). In tissue environments, the ADC value will be high (1.6–2.0 × 10^−3^ mm^2^/s) when water mobility is unrestricted, but it will be reduced (0.87–1.36 × 10^−3^ mm^2^/s) [108] when water mobility is restricted in a cell rich environment [109,110,111]. This insight has led to the use of ADC to describe the intrinsic characteristics of the tumor microenvironment, tortuosity, and cellularity. In breast cancer, based on a meta-analysis of five studies with 402 patients, ADC was moderately correlated to cellularity, suggesting that other cellular features involving the ECM, nucleic areas, stroma, parenchyma, tissue density, and cell membrane integrity may play a role in restricting water mobility in the tumor microenvironment [112]. Nevertheless, the increase in ADC is strongly associated with pCR following NAC [113,114,115,116,117,118] and may precede changes in tumor size measured by DCE-MRI [84,119,120].

**Figure 5:**
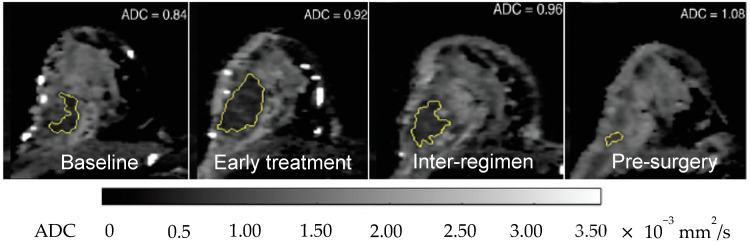
An example of an HR+/HER2− breast cancer patient who had neoadjuvant chemotherapy being monitored by MRI. The serial apparent diffusion coefficient (ADC) maps, obtained from diffusion-weighted (DW) MRI (b = 800 mm^2^/s), show an increase in value with treatment. Compared to baseline, ∆ADC = +9.5% at early treatment; +14% at inter-regimen and +29% at pre-surgery.

In a recent prospective multicenter study of 272 patients (ACRIN 6698) [121], the increase in tumor ADC values (∆ADC) were predictive of pCR at mid-treatment (AUC = 0.60; *p* = 0.017) and post-treatment (AUC = 0.61; *p* = 0.013) time points [121]. The subsequent analysis by subtype showed that ∆ADC was predictive of HR+/HER2− (*N* = 99; AUC = 0.76; *p* < 0.001) and TN tumors (*N* = 77; AUC = 0.75; *p* < 0.001) at mid-treatment, but not in HR+/HER2+ or HR−/HER2+ tumors. An example of a patient with HR+/HER2− disease being monitored by DWI is shown in Figure 5 [121]. Similar findings from a retrospective study of 225 patients also showed an improved pCR prediction in HR+/HER2− tumors when ∆ADC was adopted (*N* = 143; AUC = 0.787; *p* = 0.007) vs. Response Evaluation Criteria in Solid Tumor (RECIST) (AUC = 0.693; *p* = 0.067) [122]. When ∆ADC was combined with RECIST as predictors, further improvement in the prediction was observed in all subtypes. The additive value of ∆ADC as a covariate with the FTV metric was observed in the I-SPY 2 TRIAL with a subset of 354 patients [123]. Interestingly, while prediction improvements were found by adding ∆ADCs to the optimized FTV model at mid-treatment or post-NAC for HR+/HER2+ and TN tumors, significant improvement in predicting the HR+/HER2− subtype was found by combining pre-treatment ADC with the optimized FTV model. Given the limited benefit of chemotherapy demonstrated in the HR+/HER2− group, the use of pre-treatment ADC as a predictor would be valuable to stratify more effective treatment before the start of NAC for this breast cancer subtype.

#### 2.3.5. Magnetic Resonance Spectroscopy

^1^H-magnetic resonance spectroscopy (MRS) of total choline expression in breast lesions may be used to identify response to NAC. Metabolism and synthesis of choline-containing (tCho) compounds are increased in breast lesions, due to their role in the generation of lipid membranes and cell proliferation [124]. pCR and long-term recurrence-free survival have been linked to the reduction in tCho measured by single-voxel MRS for patients receiving NAC [125]. However, although estrogen receptor-α (ERα) may be implicated in choline metabolism [126], there is minimal evidence suggesting differentiation of tCho levels by subtype [127]. Nevertheless, additional research with larger cohorts undergoing neoadjuvant therapy with multi-voxel spectroscopy is required for assessing the utility of MRS in identifying therapy response.

In summary, technological development in breast MRI has not only improved data acquisition and image quality, but it has also yielded important volumetric (FTV), enhancement (BPE), and diffusion (ADC) markers for response to NAC. Further analyses by subtype have provided insights into their potential use to assess ER+ breast cancers. Given that DWI can be added to the existing DCE-MRI sequence with little time and cost penalty, evaluation of tumor ADC along with FTV and BPE can be easily implemented. As discussed, the sensitivity of BPE to endogenous hormone or exogenous ER modulators makes it a promising marker for ER+ breast cancer. Further studies to improve intra- and inter-reader variability, protocol standardization, quality assurance and control (QA/QC), and consensus of quantification methods are all necessary to translate qualitative and/or quantitative BPE as a marker for ER+ breast cancer.

### 2.4. Positron Emission Tomography

Positron emission tomography (PET) is a nuclear imaging technique that detects signals from two photons at 511 keV, emitted in the opposite direction from the annihilation of a positron and an electron. When the two photons are detected in coincidence, the event’s origin can be determined along the image line of response between the detector pair [128]. Technological advances in positron detection and clinical PET system designs had sparked innovation in radiochemistry to produce biologically relevant molecules for PET imaging, offering unique metabolic and biochemical information of human diseases. Indeed, since the first 30 compounds labeled with ^15^O, ^11^C, ^15^N, and ^18^F published in 1980 [129], there are now over 600 compounds at various stages of research and five approved by the FDA for human use [130].

#### 2.4.1. [^18^F]fluorodeoxyglucose

[^18^F]fluorodeoxyglucose ([^18^F]FDG) PET is the workhorse of functional imaging of cancer in clinical care today. It is used extensively for diagnosis, staging, and therapy monitoring. Similar to the glucose molecule, [^18^F]FDG is actively transported into metabolically active cells by glucose transporters and phosphorylated by hexokinase as the first step toward glycolysis. However, unlike glucose, instead of entering the glycolytic pathway for energy production, [^18^F]FDG is metabolically trapped in the cells as [^18^F]FDG-6-phosphate [131]. [^18^F]FDG is particularly sensitive to cancer cells that demonstrate an increased level of glucose transporters and hexokinase and its uptake is an imaging biomarker for all types of cancers including breast. In assessing pCR in breast cancer patients receiving NAC, several clinical studies show the value of [^18^F]FDG PET in the prediction of pathological response with relatively high sensitivity, NPV, and diagnostic accuracy [132]. In a meta-analysis of 19 studies consisting of 920 patients [133], [^18^F]FDG PET had the pooled sensitivity of 84%, specificity of 66%, PPV of 50%, NPV of 91%, diagnostic odds ratio (DOR) of 11.90, and AUC of 0.843. In a subgroup analysis focusing on timing and reduction rate cutoff, [^18^F]FDG PET at early post-treatment time points (after the first or second cycle) with a reduction of maximum standardized uptake values (ΔSUV_max_) between 55–65% were found to further improve the accuracy and correlation with pathological response. Given the relatively high sensitivity and NPV of [^18^F]FDG PET, an unfavorable result early during the NAC may signal the low likelihood of pathological response and the consideration of treatment redirection. However, owing to the relatively low specificity and PPV, favorable scans from [^18^F]FDG PET need to be interpreted with caution. Based on Dose-Schwarz’s work on assessing residual tumors using different imaging modalities [134], MRI demonstrated the highest specificity, while [^18^F]FDG PET had the highest sensitivity. Therefore, the use of [^18^F]FDG PET may complement MRI in the prediction of response to NAC and to facilitate treatment intervention to improve clinical outcomes.

In addition to SUV_max_ as a metric used in [^18^F]FDG PET, other measurements such as mean uptake (SUV_mean_), peak uptake (SUV_peak_) [135], total lesion glycolysis (TLG), metabolic tumor volume (MTV) [136], and tumor blood flow [137,138] were also represented in the literature. Importantly, the most recent PET Response Criteria for Solid Tumors (PERCIST) recommends the use of SUV_peak_ normalized to the liver SUV_mean_ [139], termed SUL_peak_, to reduce the influence of operator- and acquisition-driven noise in the SUV_max_ and SUV_mean_ measurements.

The use of [^18^F]FDG PET SUV_max_ or SUL_peak_ to stratify response in ER+/HER2− breast cancer is met with challenges. First, pre-treatment [^18^F]FDG uptake for this group is low (usually SUV_max_ < 5), making quantification of post-treatment change difficult [132]. Second, ER+/HER2− tumors have variable chemosensitivity and rarely achieve pCR in the NAC setting [140,141,142]. Finally, SUV prediction of response is more accurate at early time points during NAC, but ER+/HER2− tumors are slow to respond. Given these challenges, there is a clinical need to investigate more accurate predictors for this subgroup. In a prospective NAC study of 64 ER+/HER2− patients, Groheux et al. examined the value of different [^18^F]FDG PET measurements for prediction of early response [143]. Their findings indicated that ΔTLG, which accounts for SUV_mean_ and MTV, was more predictive than ΔSUV_max_ (AUC of 0.81 vs. 0.73), demonstrating that more studies are needed to explore alternative markers to better describe the ER+/HER2− subtype.

#### 2.4.2. [^18^F]fluoroestradiol

[^18^F]fluoroestradiol ([^18^F]FES) is a radiotracer that has been developed for PET imaging of ER status [144,145]. As an analog of estradiol, [^18^F]FES binds to ERα and serves as a surrogate marker for ERα expression [146]. Earlier clinical studies show that [^18^F]FES tumor uptake correlates with the level of ERα expression in biopsy samples by immunohistochemistry [147] and predicts the likelihood of response to endocrine treatment [148,149,150]. [^18^F]FES can detect ER+ breast cancers with high sensitivity (84%) and specificity (98%) [151]. At baseline, using an SUV cutoff ≥ 2.0, [^18^F]FES has been shown to correlate with salvage endocrine therapy response with a PPV of 50–79% and NPV of 81–88% [146,148,152,153]. When a lower cutoff was used, no patients with a baseline [^18^F]FES SUV < 1.5 had response to salvage endocrine treatment [150]. These findings underscore the value of baseline [^18^F]FES to determine treatment for ER+ breast cancer patients according to their predicted endocrine treatment responsiveness.

**Figure 6:**
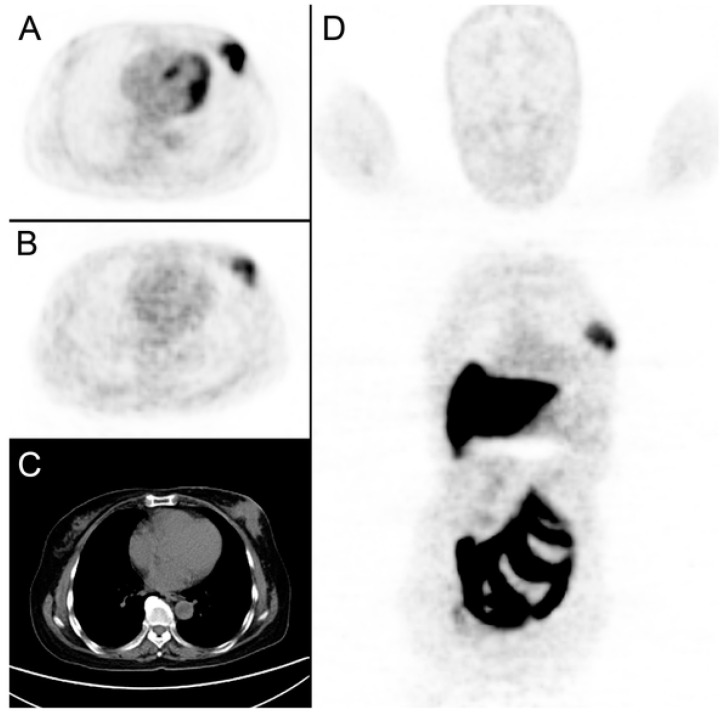
An example of a breast cancer patient receiving both [^18^F]FDG and [^18^F]FES-PET/CT scans prior to neoadjuvant chemotherapy. (**A**): A tumor was detected in left breast with [^18^F]FDG SUV_max_ at 13.51. (**B**,**D**): [^18^F]FES SUV_max_ at 4.3. (**C**): CT imaging with the tumor diameter measured at 5.3 cm.

While the predominant usage of [^18^F]FES PET was to evaluate metastatic breast cancer with salvage endocrine therapy, there is a small number of studies using [^18^F]FES PET to image the primary breast tumor in the neoadjuvant therapy paradigm [154,155] (Figure 6). In a small 18-patient cohort receiving NAC, baseline [^18^F]FES and [^18^F]FDG uptake was correlated to histopathologic response [155,156]. This study suggested [^18^F]FES and [^18^F]FDG provided similar information in ER+ patients receiving NAC. In another recent study, [^18^F]FES PET was used to evaluate neoadjuvant treatment response in post-menopausal ER+ breast cancer patients randomized to the NAC vs. the NET arm of the NEOCENT trial (*N* = 26) [157]. An important finding from this study was that patients who were [^18^F]FES-negative or -low (SUV cutoff < 7.3) at baseline achieve a higher rate of pCR in the NAC arm compared to [^18^F]FES-avid patients. None of the [^18^F]FES-negative and -low patients responded to NET. While all the patients in this study were ER+, some patients were [^18^F]FES-negative, indicating the existence of non-functional ER that may be resistant to endocrine treatment. Adequately powered prospective studies in a larger number of ER+ patients are needed to further establish the role of [^18^F]FES-PET in evaluating response to NET [158].

#### 2.4.3. Other Radiotracers

Other radiotracers that have been tested in locally advanced breast cancer include ^18^F-fluorothymidine ([^18^F]FLT), [^11^C]choline, and [^68^Ga]-labeled fibroblast activation protein inhibitors ([^68^Ga]FAPI). Like [^18^F]FES, these molecules target complementary pathways or biomarkers, potentially serving as an in vivo assay and indicator of response to specific therapeutic agents. [^18^F]FLT signal is correlated to the proliferation marker Ki-67 [159]. [^18^F]FLT uptake and irreversible retention are mediated by its phosphorylation of thymidine kinase-1, which is especially overexpressed in cells in the S-phase. Relative and absolute [^18^F]FLT SUV_max_ was only weakly predictive of pCR (AUC = 0.68) in the ACRIN 6688 trial [160]. Post-treatment [^18^F]FLT ΔSUV_max_ correlated to Ki-67 more strongly (r = 0.67, *p* < 0.0001) than baseline and absolute SUV_max_ (r = 0.29, *p* = 0.04). The weak association between pCR and [^18^F]FLT may be partially driven by the variable chemotherapy regimens and heterogeneous patient population underpinning the NAC trial. Separately, [^18^F]FLT SUV_max_ and uptake rate were found to correlate with Ki-67 in ER+ patients receiving NET [161] and NAC [162], although there was no predictive value associated to either baseline SUV_max_ or ΔSUV_max_ for stratifying response.

Lipid synthesis can be imaged by PET using [^11^C]choline. ^11^C-choline SUV_max_ is correlated to cell proliferation measured by [^18^F]FLT (r = 0.83, *p* < 0.001) [163]. There have also been preliminary studies associating [^11^C]choline uptake to response to trastuzumab in a small population (*N* = 21) of HER2+ [164] and tumor aggressiveness in ER+ patients (*N* = 32) [165]. These results are promising and warrant testing in larger cohorts receiving NAC and NET, although the use of [^11^C] may be detrimental to widespread acceptance due to the 20 min half-life and limited availability of on-site cyclotrons outside research institutions.

Cancer-associated fibroblasts (CAFs) are found abundantly in the tumor stroma. They produce growth factors and cytokines to support tumor growth [166], while enabling ECM remodeling to facilitate invasion [167,168] and creating collagen barriers against drug infiltration [169,170]. CAFs are an important source of estrogen, with high expression and activity of cancer-associated aromatase [171,172] in ER+ breast cancers. They provide nutrients for epithelial cancer cell mitochondrial activity to induce endocrine treatment resistance [173]. One distinct characteristic of CAFs is the overexpression of fibroblast activation protein-α (FAP). FAP is a type II integral membrane serine protease of the prolyl oligopeptidase family [174,175] that is highly upregulated in almost all carcinomas with low to undetectable expression in most normal tissue [176]. Based on the design of a FAP inhibitor (FAPI) [177], a collection of quinoline-based molecules labeled with [^68^Ga] have been tested for PET imaging in animals and humans [178]. In a study of 80 patients with 28 different tumors, the uptake of [^68^Ga]FAPI in breast cancer is among the highest with SUV_max_ > 12 (SUV_max, blood_ = 1.5) and tumor-to-background ratio > 6 [179]. Because of this high tumor-to-background ratio, more accurate tumor delineation is expected. Furthermore, because FAPI targets the stromal volume rather than cancer cells, imaging of FAP expression in the tumor may be more sensitive than traditional metabolic imaging of cancer cells with [^18^F]FDG [180]. These exciting new findings point to a new direction of “stroma-centric” targeting strategy in a genetically stable microenvironment that is relatively insensitive to tumor heterogeneity. Further clinical testing of [^68^Ga]FAPI in the detection and treatment monitoring of breast cancers, especially the ER+ subtype, is eagerly awaited.

So far, collective evidence suggests that functional imaging with PET offers unique information on breast tumor biology, growth, invasion, and drug resistance. [^18^F]FES PET could be a valuable tool to interrogate ER directly. [^18^F]FLT can be used in NET trials to optimize the timing for Ki67 measurements. With CAFs being a vital source of estrogen with high expression of cancer-associated aromatase, [^68^Ga]FAPI may present a new direction to assess ER+ tumors and their response to NET. Most of the radiotracers are used in breast imaging of small cohorts. However, [^18^F]FES has recently gained regulatory approval for clinical use in France and in the U.S. With this exciting development, more extensive studies are anticipated to pave the qualification of [^18^F]FES as a biomarker for ER+ breast cancer.

## 3. Conclusions

In this review article, we surveyed the current landscape of imaging techniques for assessing breast tumor response to neoadjuvant treatment. We highlighted some promising imaging markers for ER+ breast cancers. Given that each imaging modality has limitations in sensitivity and specificity, multiparametric (e.g., FTV/BPE/ADC) and multimodality (e.g., MRI/PET) approaches will likely be employed to improve the characterization of ER+ breast cancers and the prediction of response to NAC or NET. Imaging studies correlating histopathologic biomarkers such as (but is not limited to) ER status, Ki67, and PEPI score will provide valuable insight into breast tumor biology and ultimately allow for the use of imaging as a non-invasive in vivo assay for ER+ breast cancer characterization.

Recent advances in the radiomics-based analysis have demonstrated the power of transforming imaging data into multi-dimensional mineable radiomic features [181,182] that are relatable to gene expression patterns [183,184,185]. Machine-learning methods can be used to select radiomic features and build predictive models for clinical outcomes [186,187]. With the advances in high-resolution breast imaging techniques [188,189,190,191], detailed radiologic information along with consequential molecular signatures can be extracted, correlated, trained, and learned, yielding models with significant predictive and prognostic power [183,192,193,194]. Deep-learning [195] of mammographic images has been increasingly used to aid breast cancer detection [196], prediction of breast cancer risks [197,198], and identification of significant microcalcifications evaluation [199]. These new computational techniques will likely be applied to other breast imaging techniques and will play a major role in future breast cancer detection, diagnosis, and prediction of treatment outcomes.

## References

[1] Perou C.M., Sorlie T., Eisen M.B., van de Rijn M., Jeffrey S.S., Rees C.A., Pollack J.R., Ross D.T., Johnsen H., Akslen L.A. (2000). Molecular portraits of human breast tumours. Nature.

[2] Sorlie T., Perou C.M., Tibshirani R., Aas T., Geisler S., Johnsen H., Hastie T., Eisen M.B., van de Rijn M., Jeffrey S.S. (2001). Gene expression patterns of breast carcinomas distinguish tumor subclasses with clinical implications. Proc. Natl. Acad. Sci. USA.

[3] Rouzier R., Perou C.M., Symmans W.F., Ibrahim N., Cristofanilli M., Anderson K., Hess K.R., Stec J., Ayers M., Wagner P. (2005). Breast cancer molecular subtypes respond differently to preoperative chemotherapy. Clin. Cancer Res..

[4] Sotiriou C., Neo S.Y., McShane L.M., Korn E.L., Long P.M., Jazaeri A., Martiat P., Fox S.B., Harris A.L., Liu E.T. (2003). Breast cancer classification and prognosis based on gene expression profiles from a population-based study. Proc. Natl. Acad. Sci. USA.

[5] Van ‘t Veer L.J., Dai H., van de Vijver M.J., He Y.D., Hart A.A., Mao M., Peterse H.L., van der Kooy K., Marton M.J., Witteveen A.T. (2002). Gene expression profiling predicts clinical outcome of breast cancer. Nature.

[6] Carey L.A., Dees E.C., Sawyer L., Gatti L., Moore D.T., Collichio F., Ollila D.W., Sartor C.I., Graham M.L., Perou C.M. (2007). The triple negative paradox: Primary tumor chemosensitivity of breast cancer subtypes. Clin. Cancer Res..

[7] Huang E., Cheng S.H., Dressman H., Pittman J., Tsou M.H., Horng C.F., Bild A., Iversen E.S., Liao M., Chen C.M. (2003). Gene expression predictors of breast cancer outcomes. Lancet.

[8] Howlader N., Altekruse S.F., Li C.I., Chen V.W., Clarke C.A., Ries L.A., Cronin K.A. (2014). US incidence of breast cancer subtypes defined by joint hormone receptor and HER2 status. J. Natl. Cancer Inst..

[9] Anderson W.F., Chen B.E., Jatoi I., Rosenberg P.S. (2006). Effects of estrogen receptor expression and histopathology on annual hazard rates of death from breast cancer. Breast Cancer Res. Treat..

[10] Goncalves R., Ma C., Luo J., Suman V., Ellis M.J. (2012). Use of neoadjuvant data to design adjuvant endocrine therapy trials for breast cancer. Nat. Rev. Clin. Oncol..

[11] Fisher B., Bryant J., Wolmark N., Mamounas E., Brown A., Fisher E.R., Wickerham D.L., Begovic M., DeCillis A., Robidoux A. (1998). Effect of preoperative chemotherapy on the outcome of women with operable breast cancer. J. Clin. Oncol..

[12] Wolmark N., Wang J., Mamounas E., Bryant J., Fisher B. (2001). Preoperative chemotherapy in patients with operable breast cancer: Nine-year results from National Surgical Adjuvant Breast and Bowel Project B-18. J. Natl. Cancer Inst. Monogr..

[13] Esposito A., Criscitiello C., Curigliano G. (2015). Neoadjuvant Model for Testing Emerging Targeted Therapies in Breast Cancer. J. Natl. Cancer Inst. Monogr..

[14] Cortazar P., Zhang L., Untch M., Mehta K., Costantino J.P., Wolmark N., Bonnefoi H., Cameron D., Gianni L., Valagussa P. (2014). Pathological complete response and long-term clinical benefit in breast cancer: The CTNeoBC pooled analysis. Lancet.

[15] Kong X., Moran M.S., Zhang N., Haffty B., Yang Q. (2011). Meta-analysis confirms achieving pathological complete response after neoadjuvant chemotherapy predicts favourable prognosis for breast cancer patients. Eur. J. Cancer.

[16] Von Minckwitz G., Untch M., Blohmer J.U., Costa S.D., Eidtmann H., Fasching P.A., Gerber B., Eiermann W., Hilfrich J., Huober J. (2012). Definition and impact of pathologic complete response on prognosis after neoadjuvant chemotherapy in various intrinsic breast cancer subtypes. J. Clin. Oncol..

[17] Barroso-Sousa R., Silva D.D., Alessi J.V., Mano M.S. (2016). Neoadjuvant endocrine therapy in breast cancer: Current role and future perspectives. Ecancermedicalscience.

[18] Chia Y.H., Ellis M.J., Ma C.X. (2010). Neoadjuvant endocrine therapy in primary breast cancer: Indications and use as a research tool. Br. J. Cancer.

[19] Guerrero-Zotano A.L., Arteaga C.L. (2017). Neoadjuvant Trials in ER(+) Breast Cancer: A Tool for Acceleration of Drug Development and Discovery. Cancer Discov..

[20] Ueno T., Saji S., Masuda N., Kuroi K., Sato N., Takei H., Yamamoto Y., Ohno S., Yamashita H., Hisamatsu K. (2018). Impact of clinical response to neoadjuvant endocrine therapy on patient outcomes: A follow-up study of JFMC34-0601 multicentre prospective neoadjuvant endocrine trial. ESMO Open.

[21] Guidance for Industry Pathologic Complete Response in Neoadjuvant Treatment of High-Risk Early-Stage Breast Cancer: Use as an Endpoint to Support Accelerated Approval; Draft Guidance. www.fda.gov/downloads/Drugs/GuidanceComplianceRegulatoryInformation/Guidances/UCM305501.pdf.

[22] Di Cosimo S., Arpino G., Generali D. (2014). Neoadjuvant treatment of HER2 and hormone-receptor positive breast cancer—Moving beyond pathological complete response. Breast.

[23] Symmans W.F., Peintinger F., Hatzis C., Rajan R., Kuerer H., Valero V., Assad L., Poniecka A., Hennessy B., Green M. (2007). Measurement of residual breast cancer burden to predict survival after neoadjuvant chemotherapy. J. Clin. Oncol..

[24] Litton J.K., Scoggins M.E., Hess K.R., Adrada B.E., Murthy R.K., Damodaran S., DeSnyder S.M., Brewster A.M., Barcenas C.H., Valero V. (2019). Neoadjuvant Talazoparib for Patients With Operable Breast Cancer With a Germline BRCA Pathogenic Variant. J. Clin. Oncol..

[25] Pinard C., Debled M., Ben Rejeb H., Velasco V., Tunon de Lara C., Hoppe S., Richard E., Brouste V., Bonnefoi H., MacGrogan G. (2019). Residual cancer burden index and tumor-infiltrating lymphocyte subtypes in triple-negative breast cancer after neoadjuvant chemotherapy. Breast Cancer Res. Treat..

[26] Sharma P., Lopez-Tarruella S., Garcia-Saenz J.A., Khan Q.J., Gomez H.L., Prat A., Moreno F., Jerez-Gilarranz Y., Barnadas A., Picornell A.C. (2018). Pathological Response and Survival in Triple-Negative Breast Cancer Following Neoadjuvant Carboplatin plus Docetaxel. Clin. Cancer Res..

[27] Symmans W.F., Wei C., Gould R., Yu X., Zhang Y., Liu M., Walls A., Bousamra A., Ramineni M., Sinn B. (2017). Long-Term Prognostic Risk After Neoadjuvant Chemotherapy Associated With Residual Cancer Burden and Breast Cancer Subtype. J. Clin. Oncol..

[28] Cottu P., D’Hondt V., Dureau S., Lerebours F., Desmoulins I., Heudel P.E., Duhoux F.P., Levy C., Mouret-Reynier M.A., Dalenc F. (2018). Letrozole and palbociclib versus chemotherapy as neoadjuvant therapy of high-risk luminal breast cancer. Ann. Oncol..

[29] Sun X., Kaufman P.D. (2018). Ki-67: More than a proliferation marker. Chromosoma.

[30] Ellis M.J., Coop A., Singh B., Tao Y., Llombart-Cussac A., Janicke F., Mauriac L., Quebe-Fehling E., Chaudri-Ross H.A., Evans D.B. (2003). Letrozole inhibits tumor proliferation more effectively than tamoxifen independent of HER1/2 expression status. Cancer Res..

[31] Dowsett M., Smith I.E., Ebbs S.R., Dixon J.M., Skene A., Griffith C., Boeddinghaus I., Salter J., Detre S., Hills M. (2005). Short-term changes in Ki-67 during neoadjuvant treatment of primary breast cancer with anastrozole or tamoxifen alone or combined correlate with recurrence-free survival. Clin. Cancer Res..

[32] Ellis M.J., Suman V.J., Hoog J., Goncalves R., Sanati S., Creighton C.J., DeSchryver K., Crouch E., Brink A., Watson M. (2017). Ki67 Proliferation Index as a Tool for Chemotherapy Decisions During and After Neoadjuvant Aromatase Inhibitor Treatment of Breast Cancer: Results From the American College of Surgeons Oncology Group Z1031 Trial (Alliance). J. Clin. Oncol..

[33] Dowsett M., Ebbs S.R., Dixon J.M., Skene A., Griffith C., Boeddinghaus I., Salter J., Detre S., Hills M., Ashley S. (2005). Biomarker changes during neoadjuvant anastrozole, tamoxifen, or the combination: Influence of hormonal status and HER-2 in breast cancer—A study from the IMPACT trialists. J. Clin. Oncol..

[34] Rimm D.L., Leung S.C.Y., McShane L.M., Bai Y., Bane A.L., Bartlett J.M.S., Bayani J., Chang M.C., Dean M., Denkert C. (2019). An international multicenter study to evaluate reproducibility of automated scoring for assessment of Ki67 in breast cancer. Mod. Pathol..

[35] Ellis M.J., Tao Y., Luo J., A’Hern R., Evans D.B., Bhatnagar A.S., Chaudri Ross H.A., von Kameke A., Miller W.R., Smith I. (2008). Outcome prediction for estrogen receptor-positive breast cancer based on postneoadjuvant endocrine therapy tumor characteristics. J. Natl. Cancer Inst..

[36] Fowler A.M., Mankoff D.A., Joe B.N. (2017). Imaging Neoadjuvant Therapy Response in Breast Cancer. Radiology.

[37] Spring L.M., Gupta A., Reynolds K.L., Gadd M.A., Ellisen L.W., Isakoff S.J., Moy B., Bardia A. (2016). Neoadjuvant Endocrine Therapy for Estrogen Receptor-Positive Breast Cancer: A Systematic Review and Meta-analysis. JAMA Oncol..

[38] Savaridas S.L., Sim Y.T., Vinnicombe S.J., Purdie C.A., Thompson A.M., Evans A. (2019). Are baseline ultrasound and mammographic features associated with rates of pathological completes response in patients receiving neoadjuvant chemotherapy for breast cancer?. Cancers Imaging.

[39] Dialani V., Chadashvili T., Slanetz P.J. (2015). Role of Imaging in Neoadjuvant Therapy for Breast Cancer. Ann. Surg. Oncol..

[40] Weiss A., Lee K.C., Romero Y., Ward E., Kim Y., Ojeda-Fournier H., Einck J., Blair S.L. (2014). Calcifications on Mammogram Do Not Correlate with Tumor Size After Neoadjuvant Chemotherapy. Ann. Surg. Oncol..

[41] Feliciano Y., Mamtani A., Morrow M., Stempel M.M., Patil S., Jochelson M.S. (2017). Do Calcifications Seen on Mammography After Neoadjuvant Chemotherapy for Breast Cancer Always Need to Be Excised?. Ann. Surg. Oncol..

[42] Adrada B.E., Huo L., Lane D.L., Arribas E.M., Resetkova E., Yang W. (2015). Histopathologic Correlation of Residual Mammographic Microcalcifications After Neoadjuvant Chemotherapy for Locally Advanced Breast Cancer. Ann. Surg. Oncol..

[43] Moskovic E.C., Mansi J.L., King D.M., Murch C.R., Smith I.E. (1993). Mammography in the assessment of response to medical treatment of large primary breast cancer. Clin. Radiol..

[44] Haque W., Verma V., Hatch S., Suzanne Klimberg V., Brian Butler E., Teh B.S. (2018). Response rates and pathologic complete response by breast cancer molecular subtype following neoadjuvant chemotherapy. Breast Cancer Res. Treat..

[45] Tot T. (2015). Early (<10 mm) HER2-Positive Invasive Breast Carcinomas are Associated with Extensive Diffuse High-Grade DCIS: Implications for Preoperative Mapping, Extent of Surgical Intervention, and Disease-Free Survival. Ann. Surg. Oncol..

[46] Hooley R.J., Durand M.A., Philpotts L.E. (2016). Advances in Digital Breast Tomosynthesis. Am. J. Roentgenol..

[47] Park J.M., Edmund A., Franken J., Garg M., Fajardo L.L., Niklason L.T. (2007). Breast Tomosynthesis: Present Considerations and Future Applications. Radiographics.

[48] Mun H.S., Kim H.H., Shin H.J., Cha J.H., Ruppel P.L., Oh H.Y., Chae E.Y. (2013). Assessment of extent of breast cancer: Comparison between digital breast tomosynthesis and full-field digital mammography. Clin. Radiol..

[49] Park J., Chae E.Y., Cha J.H., Shin H.J., Choi W.J., Choi Y.-W., Kim H.H. (2018). Comparison of mammography, digital breast tomosynthesis, automated breast ultrasound, magnetic resonance imaging in evaluation of residual tumor after neoadjuvant chemotherapy. Eur. J. Radiol..

[50] Richman I., Hoag J.R., Busch S., Gross C.P. (2018). Early adoption of digital breast tomosynthesis in the United States. J. Clin. Oncol..

[51] Puong S., Bouchevreau X., Patoureaux F., Iordache R., Muller S. Dual-energy contrast enhanced digital mammography using a new approach for breast tissue canceling. Proceedings of the Medical Imaging.

[52] Alexander S., Dulku G., Hashoul S., Taylor D.B. (2019). Practical uses of contrast-enhanced spectral mammography in daily work: A pictorial review. J. Med. Imaging Radiat Oncol..

[53] Dromain C., Balleyguier C., Adler G., Garbay J.R., Delaloge S. (2009). Contrast-enhanced digital mammography. Eur. J. Radiol..

[54] James J.J., Tennant S.L. (2018). Contrast-enhanced spectral mammography (CESM). Clin. Radiol..

[55] Iotti V., Ravaioli S., Vacondio R., Coriani C., Caffarri S., Sghedoni R., Nitrosi A., Ragazzi M., Gasparini E., Masini C. (2017). Contrast-enhanced spectral mammography in neoadjuvant chemotherapy monitoring: A comparison with breast magnetic resonance imaging. Breast Cancer Res..

[56] Patel B.K., Hilal T., Covington M., Zhang N., Kosiorek H.E., Lobbes M., Northfelt D.W., Pockaj B.A. (2018). Contrast-Enhanced Spectral Mammography is Comparable to MRI in the Assessment of Residual Breast Cancer Following Neoadjuvant Systemic Therapy. Ann. Surg. Oncol..

[57] Bosch A.M., Kessels A.G.H., Beets G.L., Rupa J.D., Koster D., van Engelshoven J.M.A., von Meyenfeldt M.F. (2003). Preoperative estimation of the pathological breast tumour size by physical examination, mammography and ultrasound: A prospective study on 105 invasive tumours. Eur. J. Radiol..

[58] Keune J.D., Jeffe D.B., Schootman M., Hoffman A., Gillanders W.E., Aft R.L. (2010). Accuracy of ultrasonography and mammography in predicting pathologic response after neoadjuvant chemotherapy for breast cancer. Am. J. Surg..

[59] Candelaria R.P., Bassett R.L., Symmans W.F., Ramineni M., Moulder S.L., Kuerer H.M., Thompson A.M., Yang W.T. (2017). Performance of Mid-Treatment Breast Ultrasound and Axillary Ultrasound in Predicting Response to Neoadjuvant Chemotherapy by Breast Cancer Subtype. Oncologist.

[60] Rashmi S., Kamala S., Murthy S.S., Kotha S., Rao Y.S., Chaudhary K.V. (2018). Predicting the molecular subtype of breast cancer based on mammography and ultrasound findings. Indian J. Radiol. Imaging.

[61] Vane M.L.G., van Nijnatten T.J.A., Nelemans P.J., Lobbes M.B.I., van Roozendaal L.M., Kooreman L.F.S., Keymeulen K.B.M.I., Smidt M.L., Schipper R.J. (2019). Does the subtype of breast cancer affect the diagnostic performance of axillary ultrasound for nodal staging in breast cancer patients?. Eur. J. Surg. Oncol..

[62] Lee S.C., Grant E., Sheth P., Garcia A.A., Desai B., Ji L., Groshen S., Hwang D., Yamashita M., Hovanessian-Larsen L. (2017). Accuracy of Contrast-Enhanced Ultrasound Compared With Magnetic Resonance Imaging in Assessing the Tumor Response After Neoadjuvant Chemotherapy for Breast Cancer. J. Ultrasound Med..

[63] Jia K., Li L., Wu X.J., Hao M.J., Xue H.Y. (2019). Contrast-enhanced ultrasound for evaluating the pathologic response of breast cancer to neoadjuvant chemotherapy: A meta-analysis. Medicine.

[64] Chen L., Yang Q., Bao J., Liu D., Huang X., Wang J. (2017). Direct comparison of PET/CT and MRI to predict the pathological response to neoadjuvant chemotherapy in breast cancer: A meta-analysis. Sci. Rep..

[65] Levental K.R., Yu H., Kass L., Lakins J.N., Egeblad M., Erler J.T., Fong S.F., Csiszar K., Giaccia A., Weninger W. (2009). Matrix crosslinking forces tumor progression by enhancing integrin signaling. Cell.

[66] Youk J.H., Gweon H.M., Son E.J. (2017). Shear-wave elastography in breast ultrasonography: The state of the art. Ultrasonography.

[67] Evans A., Armstrong S., Whelehan P., Thomson K., Rauchhaus P., Purdie C., Jordan L., Jones L., Thompson A., Vinnicombe S. (2013). Can shear-wave elastography predict response to neoadjuvant chemotherapy in women with invasive breast cancer?. Br. J. Cancer.

[68] Lee S.H., Chang J.M., Han W., Moon H.G., Koo H.R., Gweon H.M., Kim W.H., Noh D.Y., Moon W.K. (2015). Shear-Wave Elastography for the Detection of Residual Breast Cancer After Neoadjuvant Chemotherapy. Ann. Surg. Oncol..

[69] Marinovich M.L., Houssami N., Macaskill P., Sardanelli F., Irwig L., Mamounas E.P., von Minckwitz G., Brennan M.E., Ciatto S. (2013). Meta-analysis of magnetic resonance imaging in detecting residual breast cancer after neoadjuvant therapy. J. Natl. Cancer Inst..

[70] Martincich L., Montemurro F., De Rosa G., Marra V., Ponzone R., Cirillo S., Gatti M., Biglia N., Sarotto I., Sismondi P. (2004). Monitoring response to primary chemotherapy in breast cancer using dynamic contrast-enhanced magnetic resonance imaging. Breast Cancer Res. Treat..

[71] Partridge S.C., Gibbs J.E., Lu Y., Esserman L.J., Tripathy D., Wolverton D.S., Rugo H.S., Hwang E.S., Ewing C.A., Hylton N.M. (2005). MRI measurements of breast tumor volume predict response to neoadjuvant chemotherapy and recurrence-free survival. AJR Am. J. Roentgenol..

[72] Kim S.-Y., Cho N., Park I.-A., Kwon B.R., Shin S.U., Kim S.Y., Lee S.H., Chang J.M., Moon W.K. (2018). Dynamic Contrast-enhanced Breast MRI for Evaluating Residual Tumor Size after Neoadjuvant Chemotherapy. Radiology.

[73] Wu W.-P., Wu H.-K., Chen C.-J., Lee C.-W., Chen S.-T., Chen D.-R., Chou C.-T., Mok C.W., Lai H.-W. (2019). Higher underestimation of tumour size post-neoadjuvant chemotherapy with breast magnetic resonance imaging (MRI)—A concordance comparison cohort analysis. PLoS ONE.

[74] Arlinghaus L.R., Li X., Levy M., Smith D., Welch E.B., Gore J.C., Yankeelov T.E. (2010). Current and future trends in magnetic resonance imaging assessments of the response of breast tumors to neoadjuvant chemotherapy. J. Oncol..

[75] Lee J., Kim S.H., Kang B.J. (2018). Pretreatment prediction of pathologic complete response to neoadjuvant chemotherapy in breast cancer: Perfusion metrics of dynamic contrast enhanced MRI. Sci. Rep..

[76] Tudorica A., Oh K.Y., Chui S.Y.C., Roy N., Troxell M.L., Naik A., Kemmer K.A., Chen Y., Holtorf M.L., Afzal A. (2016). Early Prediction and Evaluation of Breast Cancer Response to Neoadjuvant Chemotherapy Using Quantitative DCE-MRI. Transl. Oncol..

[77] Loo C.E., Teertstra H.J., Rodenhuis S., van de Vijver M.J., Hannemann J., Muller S.H., Peeters M.-J.V., Gilhuijs K.G.A. (2008). Dynamic Contrast-Enhanced MRI for Prediction of Breast Cancer Response to Neoadjuvant Chemotherapy: Initial Results. Am. J. Roentgenol..

[78] Marinovich M.L., Sardanelli F., Ciatto S., Mamounas E., Brennan M., Macaskill P., Irwig L., von Minckwitz G., Houssami N. (2012). Early prediction of pathologic response to neoadjuvant therapy in breast cancer: Systematic review of the accuracy of MRI. Breast.

[79] Partridge S.C., Heumann E.J., Hylton N.M. (1999). Semi-automated analysis for MRI of breast tumors. Stud. Health Technol. Inform..

[80] Hylton N.M., Blume J.D., Bernreuter W.K., Pisano E.D., Rosen M.A., Morris E.A., Weatherall P.T., Lehman C.D., Newstead G.M., Polin S. (2012). Locally advanced breast cancer: MR imaging for prediction of response to neoadjuvant chemotherapy—Results from ACRIN 6657/I-SPY TRIAL. Radiology.

[81] Hylton N.M., Gatsonis C.A., Rosen M.A., Lehman C.D., Newitt D.C., Partridge S.C., Bernreuter W.K., Pisano E.D., Morris E.A., Weatherall P.T. (2016). Neoadjuvant Chemotherapy for Breast Cancer: Functional Tumor Volume by MR Imaging Predicts Recurrence-free Survival-Results from the ACRIN 6657/CALGB 150007 I-SPY 1 TRIAL. Radiology.

[82] Lo W.C., Li W., Jones E.F., Newitt D.C., Kornak J., Wilmes L.J., Esserman L.J., Hylton N.M. (2016). Effect of Imaging Parameter Thresholds on MRI Prediction of Neoadjuvant Chemotherapy Response in Breast Cancer Subtypes. PLoS ONE.

[83] Li W., Arasu V., Newitt D.C., Jones E.F., Wilmes L., Gibbs J., Kornak J., Joe B.N., Esserman L.J., Hylton N.M. (2016). Effect of MR Imaging Contrast Thresholds on Prediction of Neoadjuvant Chemotherapy Response in Breast Cancer Subtypes: A Subgroup Analysis of the ACRIN 6657/I-SPY 1 TRIAL. Tomography.

[84] Pickles M.D., Gibbs P., Lowry M., Turnbull L.W. (2006). Diffusion changes precede size reduction in neoadjuvant treatment of breast cancer. Magn. Reson. Imaging.

[85] Ah-See M.L., Makris A., Taylor N.J., Harrison M., Richman P.I., Burcombe R.J., Stirling J.J., d’Arcy J.A., Collins D.J., Pittam M.R. (2008). Early changes in functional dynamic magnetic resonance imaging predict for pathologic response to neoadjuvant chemotherapy in primary breast cancer. Clin. Cancer Res..

[86] De Bazelaire C., Calmon R., Thomassin I., Brunon C., Hamy A.S., Fournier L., Balvay D., Espie M., Siauve N., Clement O. (2011). Accuracy of perfusion MRI with high spatial but low temporal resolution to assess invasive breast cancer response to neoadjuvant chemotherapy: A retrospective study. BMC Cancer.

[87] Drisis S., Metens T., Ignatiadis M., Stathopoulos K., Chao S.L., Lemort M. (2016). Quantitative DCE-MRI for prediction of pathological complete response following neoadjuvant treatment for locally advanced breast cancer: The impact of breast cancer subtypes on the diagnostic accuracy. Eur. Radiol..

[88] Li S.P., Makris A., Beresford M.J., Taylor N.J., Ah-See M.L., Stirling J.J., d’Arcy J.A., Collins D.J., Kozarski R., Padhani A.R. (2011). Use of dynamic contrast-enhanced MR imaging to predict survival in patients with primary breast cancer undergoing neoadjuvant chemotherapy. Radiology.

[89] Li X., Kang H., Arlinghaus L.R., Abramson R.G., Chakravarthy A.B., Abramson V.G., Farley J., Sanders M., Yankeelov T.E. (2014). Analyzing Spatial Heterogeneity in DCE- and DW-MRI Parametric Maps to Optimize Prediction of Pathologic Response to Neoadjuvant Chemotherapy in Breast Cancer. Transl. Oncol..

[90] O’Flynn E.A., Collins D., D’Arcy J., Schmidt M., de Souza N.M. (2016). Multi-parametric MRI in the early prediction of response to neo-adjuvant chemotherapy in breast cancer: Value of non-modelled parameters. Eur. J. Radiol..

[91] Yu Y., Jiang Q., Miao Y., Li J., Bao S., Wang H., Wu C., Wang X., Zhu J., Zhong Y. (2010). Quantitative Analysis of Clinical Dynamic Contrast-enhanced MR Imaging for Evaluating Treatment Response in Human Breast Cancer. Radiology.

[92] Jun W., Cong W., Xianxin X., Daqing J. (2019). Meta-Analysis of Quantitative Dynamic Contrast-Enhanced MRI for the Assessment of Neoadjuvant Chemotherapy in Breast Cancer. Am. Surg..

[93] Jansen S.A., Lin V.C., Giger M.L., Li H., Karczmar G.S., Newstead G.M. (2011). Normal parenchymal enhancement patterns in women undergoing MR screening of the breast. Eur. Radiol..

[94] Kuhl C.K., Bieling H.B., Gieseke J., Kreft B.P., Sommer T., Lutterbey G., Schild H.H. (1997). Healthy premenopausal breast parenchyma in dynamic contrast-enhanced MR imaging of the breast: Normal contrast medium enhancement and cyclical-phase dependency. Radiology.

[95] Muller-Schimpfle M., Ohmenhauser K., Stoll P., Dietz K., Claussen C.D. (1997). Menstrual cycle and age: Influence on parenchymal contrast medium enhancement in MR imaging of the breast. Radiology.

[96] Sung J.S., Corben A.D., Brooks J.D., Edelweiss M., Keating D.M., Lin C., Morris E.A., Patel P., Robson M., Woods M. (2018). Histopathologic characteristics of background parenchymal enhancement (BPE) on breast MRI. Breast Cancer Res. Treat..

[97] Li T., Sun L., Miller N., Nicklee T., Woo J., Hulse-Smith L., Tsao M.S., Khokha R., Martin L., Boyd N. (2005). The association of measured breast tissue characteristics with mammographic density and other risk factors for breast cancer. Cancer Epidemiol. Biomark. Prev..

[98] Morris E.A., Comstock C.E., Lee C.H. (2013). ACR BI-RADS^®^ Magnetic Resonance Imaging. ACR BI-RADS^®^ Atlas, Breast Imaging Reporting and Data System.

[99] DeMartini W.B., Liu F., Peacock S., Eby P.R., Gutierrez R.L., Lehman C.D. (2012). Background Parenchymal Enhancement on Breast MRI: Impact on Diagnostic Performance. Am. J. Roentgenol..

[100] Liao G.J., Henze Bancroft L.C., Strigel R.M., Chitalia R.D., Kontos D., Moy L., Partridge S.C., Rahbar H. (2020). Background parenchymal enhancement on breast MRI: A comprehensive review. J. Magn. Reson. Imaging.

[101] King V., Brooks J.D., Bernstein J.L., Reiner A.S., Pike M.C., Morris E.A. (2011). Background parenchymal enhancement at breast MR imaging and breast cancer risk. Radiology.

[102] Preibsch H., Wanner L., Bahrs S.D., Wietek B.M., Siegmann-Luz K.C., Oberlecher E., Hahn M., Staebler A., Nikolaou K., Wiesinger B. (2016). Background parenchymal enhancement in breast MRI before and after neoadjuvant chemotherapy: Correlation with tumour response. Eur. Radiol..

[103] Chen J.H., Yu H.J., Hsu C., Mehta R.S., Carpenter P.M., Su M.Y. (2015). Background Parenchymal Enhancement of the Contralateral Normal Breast: Association with Tumor Response in Breast Cancer Patients Receiving Neoadjuvant Chemotherapy. Transl. Oncol..

[104] Oh S.J., Chae E.Y., Cha J.H., Shin H.J., Choi W.J., Kim H.H. (2018). Relationship between background parenchymal enhancement on breast MRI and pathological tumor response in breast cancer patients receiving neoadjuvant chemotherapy. Br. J. Radiol..

[105] You C., Gu Y., Peng W., Li J., Shen X., Liu G., Peng W. (2018). Decreased background parenchymal enhancement of the contralateral breast after two cycles of neoadjuvant chemotherapy is associated with tumor response in HER2-positive breast cancer. Acta Radiol..

[106] Ragusi M., Loo C.E., van der Velden B.H., Wesseling J., Beets-Tan R.G., Elias M.S.G., Gilhuijs K.G. Change in Contralateral Parenchymal Enhancement during Neoadjuvant Endocrine Treatment is Associated with Tumor Response in Unilateral ER+/HER2- Breast Cancer Patients. Proceedings of the 105th Radiological Society of North America Annual Meeting.

[107] Hilal T., Covington M., Kosiorek H.E., Zwart C., Ocal I.T., Pockaj B.A., Northfelt D.W., Patel B.K. (2018). Breast MRI phenotype and background parenchymal enhancement may predict tumor response to neoadjuvant endocrine therapy. Breast J..

[108] Partridge S.C., McDonald E.S. (2013). Diffusion weighted magnetic resonance imaging of the breast: Protocol optimization, interpretation, and clinical applications. Magn. Reson. Imaging Clin. N. Am..

[109] Chenevert T.L., Meyer C.R., Moffat B.A., Rehemtulla A., Mukherji S.K., Gebarski S.S., Quint D.J., Robertson P.L., Lawrence T.S., Junck L. (2002). Diffusion MRI: A New Strategy for Assessment of Cancer Therapeutic Efficacy. Mol. Imaging.

[110] Ross B.D., Moffat B.A., Lawrence T.S., Mukherji S.K., Gebarski S.S., Quint D.J., Johnson T.D., Junck L., Robertson P.L., Muraszko K.M. (2003). Evaluation of Cancer Therapy Using Diffusion Magnetic Resonance Imaging. Mol. Cancer Ther..

[111] Padhani A.R., Koh D.M. (2011). Diffusion MR imaging for monitoring of treatment response. Magn. Reson. Imaging Clin. N. Am..

[112] Surov A., Meyer H.J., Wienke A. (2017). Correlation between apparent diffusion coefficient (ADC) and cellularity is different in several tumors: A meta-analysis. Oncotarget.

[113] Sharma U., Danishad K.K., Seenu V., Jagannathan N.R. (2009). Longitudinal study of the assessment by MRI and diffusion-weighted imaging of tumor response in patients with locally advanced breast cancer undergoing neoadjuvant chemotherapy. NMR Biomed..

[114] Park S.H., Moon W.K., Cho N., Song I.C., Chang J.M., Park I.A., Han W., Noh D.Y. (2010). Diffusion-weighted MR imaging: Pretreatment prediction of response to neoadjuvant chemotherapy in patients with breast cancer. Radiology.

[115] Fangberget A., Nilsen L.B., Hole K.H., Holmen M.M., Engebraaten O., Naume B., Smith H.J., Olsen D.R., Seierstad T. (2011). Neoadjuvant chemotherapy in breast cancer-response evaluation and prediction of response to treatment using dynamic contrast-enhanced and diffusion-weighted MR imaging. Eur. Radiol..

[116] Li X.R., Cheng L.Q., Liu M., Zhang Y.J., Wang J.D., Zhang A.L., Song X., Li J., Zheng Y.Q., Liu L. (2012). DW-MRI ADC values can predict treatment response in patients with locally advanced breast cancer undergoing neoadjuvant chemotherapy. Med. Oncol..

[117] Park S.H., Moon W.K., Cho N., Chang J.M., Im S.A., Park I.A., Kang K.W., Han W., Noh D.Y. (2012). Comparison of diffusion-weighted MR imaging and FDG PET/CT to predict pathological complete response to neoadjuvant chemotherapy in patients with breast cancer. Eur. Radiol..

[118] Leong K.M., Lau P., Ramadan S. (2015). Utilisation of MR spectroscopy and diffusion weighted imaging in predicting and monitoring of breast cancer response to chemotherapy. J. Med. Imaging Radiat. Oncol..

[119] Shin H.J., Kim S.H., Lee H.J., Gong G., Baek S., Chae E.Y., Choi W.J., Cha J.H., Kim H.H. (2016). Tumor apparent diffusion coefficient as an imaging biomarker to predict tumor aggressiveness in patients with estrogen-receptor-positive breast cancer. NMR Biomed..

[120] Pereira N.P., Curi C., Osorio C., Marques E.F., Makdissi F.B., Pinker K., Bitencourt A.G.V. (2019). Diffusion-Weighted Magnetic Resonance Imaging of Patients with Breast Cancer Following Neoadjuvant Chemotherapy Provides Early Prediction of Pathological Response—A Prospective Study. Sci. Rep..

[121] Partridge S.C., Zhang Z., Newitt D.C., Gibbs J.E., Chenevert T.L., Rosen M.A., Bolan P.J., Marques H.S., Romanoff J., Cimino L. (2018). Diffusion-weighted MRI Findings Predict Pathologic Response in Neoadjuvant Treatment of Breast Cancer: The ACRIN 6698 Multicenter Trial. Radiology.

[122] Bufi E., Belli P., Di Matteo M., Terribile D., Franceschini G., Nardone L., Petrone G., Bonomo L. (2014). Effect of breast cancer phenotype on diagnostic performance of MRI in the prediction to response to neoadjuvant treatment. Eur. J. Radiol..

[123] Li W., Newitt D.C., Wilmes L.J., Jones E.F., Arasu V., Gibbs J., La Yun B., Li E., Partridge S.C., Kornak J. (2019). Additive value of diffusion-weighted MRI in the I-SPY 2 TRIAL. J. Magn. Reson. Imaging JMRI.

[124] Haukaas H.T., Euceda R.L., Giskeødegård F.G., Bathen F.T. (2017). Metabolic Portraits of Breast Cancer by HR MAS MR Spectroscopy of Intact Tissue Samples. Metabolites.

[125] Cao M.D., Sitter B., Bathen T.F., Bofin A., Lønning P.E., Lundgren S., Gribbestad I.S. (2012). Predicting long-term survival and treatment response in breast cancer patients receiving neoadjuvant chemotherapy by MR metabolic profiling. NMR Biomed..

[126] Jia M., Andreassen T., Jensen L., Bathen T.F., Sinha I., Gao H., Zhao C., Haldosen L.A., Cao Y., Girnita L. (2016). Estrogen Receptor alpha Promotes Breast Cancer by Reprogramming Choline Metabolism. Cancer Res..

[127] Sah R.G., Sharma U., Parshad R., Seenu V., Mathur S.R., Jagannathan N.R. (2012). Association of estrogen receptor, progesterone receptor, and human epidermal growth factor receptor 2 status with total choline concentration and tumor volume in breast cancer patients: An MRI and in vivo proton MRS study. Magn. Reson. Med..

[128] National Research Council (US) and Institute of Medicine (US) Committee on the Mathematics and Physics of Emerging Dynamic Biomedical Imaging (1996). Positron Emission Tomography. Mathematics and Physics of Emerging Biomedical Imaging.

[129] Ter-Pogossian M.M., Raichle M.E., Sobel B.E. (1980). Positron-Emission Tomography. Sci. Am..

[130] Molecular Imaging and Contrast Agent Database (MICAD) [Internet] Bethesda (MD): National Center for Biotechnology Information (USA); 2004–2013. About MICAD. https://www.ncbi.nlm.nih.gov/books/NBK5923/.

[131] Mankoff D.A., Eary J.F., Link J.M., Muzi M., Rajendran J.G., Spence A.M., Krohn K.A. (2007). Tumor-specific positron emission tomography imaging in patients: [18F] fluorodeoxyglucose and beyond. Clin. Cancer Res..

[132] Groheux D., Mankoff D., Espie M., Hindie E. (2016). (1)(8)F-FDG PET/CT in the early prediction of pathological response in aggressive subtypes of breast cancer: Review of the literature and recommendations for use in clinical trials. Eur. J. Nucl. Med. Mol. Imaging.

[133] Wang Y., Zhang C., Liu J., Huang G. (2012). Is 18F-FDG PET accurate to predict neoadjuvant therapy response in breast cancer? A meta-analysis. Breast Cancer Res. Treat..

[134] Dose-Schwarz J., Tiling R., Avril-Sassen S., Mahner S., Lebeau A., Weber C., Schwaiger M., Janicke F., Untch M., Avril N. (2010). Assessment of residual tumour by FDG-PET: Conventional imaging and clinical examination following primary chemotherapy of large and locally advanced breast cancer. Br. J. Cancer.

[135] Avril S., Muzic R.F., Plecha D., Traughber B.J., Vinayak S., Avril N. (2016). ¹⁸F-FDG PET/CT for Monitoring of Treatment Response in Breast Cancer. J. Nucl. Med..

[136] Gallamini A., Zwarthoed C., Borra A. (2014). Positron Emission Tomography (PET) in Oncology. Cancers.

[137] Dunnwald L.K., Gralow J.R., Ellis G.K., Livingston R.B., Linden H.M., Specht J.M., Doot R.K., Lawton T.J., Barlow W.E., Kurland B.F. (2008). Tumor metabolism and blood flow changes by positron emission tomography: Relation to survival in patients treated with neoadjuvant chemotherapy for locally advanced breast cancer. J. Clin. Oncol..

[138] Mankoff D.A., Dunnwald L.K., Gralow J.R., Ellis G.K., Schubert E.K., Tseng J., Lawton T.J., Linden H.M., Livingston R.B. (2003). Changes in blood flow and metabolism in locally advanced breast cancer treated with neoadjuvant chemotherapy. J. Nucl. Med..

[139] O J.H., Lodge M.A., Wahl R.L. (2016). Practical PERCIST: A Simplified Guide to PET Response Criteria in Solid Tumors 1.0. Radiology.

[140] Bhargava R., Beriwal S., Dabbs D.J., Ozbek U., Soran A., Johnson R.R., Brufsky A.M., Lembersky B.C., Ahrendt G.M. (2010). Immunohistochemical surrogate markers of breast cancer molecular classes predicts response to neoadjuvant chemotherapy: A single institutional experience with 359 cases. Cancer.

[141] Lips E.H., Mulder L., de Ronde J.J., Mandjes I.A., Vincent A., Vrancken Peeters M.T., Nederlof P.M., Wesseling J., Rodenhuis S. (2012). Neoadjuvant chemotherapy in ER+ HER2- breast cancer: Response prediction based on immunohistochemical and molecular characteristics. Breast Cancer Res. Treat..

[142] Semiglazov V.F., Semiglazov V.V., Dashyan G.A., Ziltsova E.K., Ivanov V.G., Bozhok A.A., Melnikova O.A., Paltuev R.M., Kletzel A., Berstein L.M. (2007). Phase 2 randomized trial of primary endocrine therapy versus chemotherapy in postmenopausal patients with estrogen receptor-positive breast cancer. Cancer.

[143] Groheux D., Hatt M., Hindie E., Giacchetti S., de Cremoux P., Lehmann-Che J., Martineau A., Marty M., Cuvier C., Cheze-Le Rest C. (2013). Estrogen receptor-positive/human epidermal growth factor receptor 2-negative breast tumors: Early prediction of chemosensitivity with (18)F-fluorodeoxyglucose positron emission tomography/computed tomography during neoadjuvant chemotherapy. Cancer.

[144] Kiesewetter D.O., Kilbourn M.R., Landvatter S.W., Heiman D.F., Katzenellenbogen J.A., Welch M.J. (1984). Preparation of four fluorine- 18-labeled estrogens and their selective uptakes in target tissues of immature rats. J. Nucl. Med..

[145] Katzenellenbogen J.A., Welch M.J., Dehdashti F. (1997). The development of estrogen and progestin radiopharmaceuticals for imaging breast cancer. Anticancer Res..

[146] Dehdashti F., Mortimer J.E., Siegel B.A., Griffeth L.K., Bonasera T.J., Fusselman M.J., Detert D.D., Cutler P.D., Katzenellenbogen J.A., Welch M.J. (1995). Positron tomographic assessment of estrogen receptors in breast cancer: Comparison with FDG-PET and in vitro receptor assays. J. Nucl. Med..

[147] Peterson L.M., Mankoff D.A., Lawton T., Yagle K., Schubert E.K., Stekhova S., Gown A., Link J.M., Tewson T., Krohn K.A. (2008). Quantitative imaging of estrogen receptor expression in breast cancer with PET and 18F-fluoroestradiol. J. Nucl. Med..

[148] Mortimer J.E., Dehdashti F., Siegel B.A., Katzenellenbogen J.A., Fracasso P., Welch M.J. (1996). Positron emission tomography with 2-[18F]Fluoro-2-deoxy-D-glucose and 16alpha-[18F]fluoro-17beta-estradiol in breast cancer: Correlation with estrogen receptor status and response to systemic therapy. Clin. Cancer Res..

[149] Mortimer J.E., Dehdashti F., Siegel B.A., Trinkaus K., Katzenellenbogen J.A., Welch M.J. (2001). Metabolic flare: Indicator of hormone responsiveness in advanced breast cancer. J. Clin. Oncol..

[150] Linden H.M., Stekhova S.A., Link J.M., Gralow J.R., Livingston R.B., Ellis G.K., Petra P.H., Peterson L.M., Schubert E.K., Dunnwald L.K. (2006). Quantitative fluoroestradiol positron emission tomography imaging predicts response to endocrine treatment in breast cancer. J. Clin. Oncol..

[151] Van Kruchten M., de Vries E.G.E., Brown M., de Vries E.F.J., Glaudemans A.W.J.M., Dierckx R.A.J.O., Schröder C.P., Hospers G.A.P. (2013). PET imaging of oestrogen receptors in patients with breast cancer. Lancet Oncol..

[152] Fowler A.M., Clark A.S., Katzenellenbogen J.A., Linden H.M., Dehdashti F. (2016). Imaging Diagnostic and Therapeutic Targets: Steroid Receptors in Breast Cancer. J. Nucl. Med..

[153] Liao G.J., Clark A.S., Schubert E.K., Mankoff D.A. (2016). 18F-Fluoroestradiol PET: Current Status and Potential Future Clinical Applications. J. Nucl. Med..

[154] Sun Y., Yang Z., Zhang Y., Xue J., Wang M., Shi W., Zhu B., Hu S., Yao Z., Pan H. (2015). The Preliminary Study of 16α-[18F]fluoroestradiol PET/CT in Assisting the Individualized Treatment Decisions of Breast Cancer Patients. PLoS ONE.

[155] Yang Z., Sun Y., Xue J., Yao Z., Xu J., Cheng J., Shi W., Zhu B., Zhang Y., Zhang Y. (2013). Can Positron Emission Tomography/Computed Tomography with the Dual Tracers Fluorine-18 Fluoroestradiol and Fluorodeoxyglucose Predict Neoadjuvant Chemotherapy Response of Breast Cancer?—A Pilot Study. PLoS ONE.

[156] Sataloff D.M., Mason B.A., Prestipino A.J., Seinige U.L., Lieber C.P., Baloch Z. (1995). Pathologic response to induction chemotherapy in locally advanced carcinoma of the breast: A determinant of outcome. J. Am. Coll. Surg..

[157] Chae S.Y., Kim S.-B., Ahn S.H., Kim H.O., Yoon D.H., Ahn J.-H., Jung K.H., Han S., Oh S.J., Lee S.J. (2017). A Randomized Feasibility Study of 18F-Fluoroestradiol PET to Predict Pathologic Response to Neoadjuvant Therapy in Estrogen Receptor–Rich Postmenopausal Breast Cancer. J. Nucl. Med..

[158] Fowler A.M., Linden H.M. (2017). Functional Estrogen Receptor Imaging Before Neoadjuvant Therapy for Primary Breast Cancer. J. Nucl. Med..

[159] Mankoff D.A., Shields A.F., Krohn K.A. (2005). PET imaging of cellular proliferation. Radiol. Clin. N Am..

[160] Kostakoglu L., Duan F., Idowu M.O., Jolles P.R., Bear H.D., Muzi M., Cormack J., Pryma D.A., Specht J.M., Hovanessian Larsen L. (2014). Phase II study of 3′-deoxy-3′-18F fluorothymidine PET/CT (FLT-PET) in the assessment of early response in locally advanced breast cancer (LABC): Preliminary results of ACRIN 6688. J. Clin. Oncol..

[161] Roberts T.K., Peterson L., Kurland B., Novakova A., Shields A., Doot R.K., Schubert E.K., Gadi V.K., Specht J.M., Gralow J. (2016). Use of serial 18F-Fluorothymidine (FLT) PET and Ki-67 to predict response to aromatase inhibitors (AI) in women with ER+ breast cancer. J. Clin. Oncol..

[162] Woolf D.K., Beresford M., Li S.P., Dowsett M., Sanghera B., Wong W.L., Sonoda L., Detre S., Amin V., Ah-See M.L. (2014). Evaluation of FLT-PET-CT as an imaging biomarker of proliferation in primary breast cancer. Br. J. Cancer.

[163] Contractor K.B., Kenny L.M., Stebbing J., Challapalli A., Al-Nahhas A., Palmieri C., Shousha S., Lewis J.S., Hogben K., De Nguyen Q. (2011). Biological basis of [11C]choline-positron emission tomography in patients with breast cancer: Comparison with [18F]fluorothymidine positron emission tomography. Nucl. Med. Commun..

[164] Kenny L.M., Contractor K.B., Hinz R., Stebbing J., Palmieri C., Jiang J., Shousha S., Al-Nahhas A., Coombes R.C., Aboagye E.O. (2010). Reproducibility of [11C]choline-positron emission tomography and effect of trastuzumab. Clin. Cancer Res..

[165] Contractor K.B., Kenny L.M., Stebbing J., Al-Nahhas A., Palmieri C., Sinnett D., Lewis J.S., Hogben K., Osman S., Shousha S. (2009). [^11^C]Choline Positron Emission Tomography in Estrogen Receptor–Positive Breast Cancer. Clin. Cancer Res..

[166] Luo H., Tu G., Liu Z., Liu M. (2015). Cancer-associated fibroblasts: A multifaceted driver of breast cancer progression. Cancer Lett..

[167] Brentnall T.A. (2012). Arousal of cancer-associated stromal fibroblasts: Palladin-activated fibroblasts promote tumor invasion. Cell Adh. Migr..

[168] Santi A., Kugeratski F.G., Zanivan S. (2018). Cancer Associated Fibroblasts: The Architects of Stroma Remodeling. Proteomics.

[169] Kalluri R. (2016). The biology and function of fibroblasts in cancer. Nat. Rev. Cancer.

[170] Amornsupak K., Insawang T., Thuwajit P., O-Charoenrat P., Eccles S.A., Thuwajit C. (2014). Cancer-associated fibroblasts induce high mobility group box 1 and contribute to resistance to doxorubicin in breast cancer cells. BMC Cancer.

[171] Miki Y., Suzuki T., Tazawa C., Yamaguchi Y., Kitada K., Honma S., Moriya T., Hirakawa H., Evans D.B., Hayashi S. (2007). Aromatase localization in human breast cancer tissues: Possible interactions between intratumoral stromal and parenchymal cells. Cancer Res..

[172] Yamaguchi Y., Takei H., Suemasu K., Kobayashi Y., Kurosumi M., Harada N., Hayashi S. (2005). Tumor-stromal interaction through the estrogen-signaling pathway in human breast cancer. Cancer Res..

[173] Martinez-Outschoorn U.E., Goldberg A.F., Lin Z., Ko Y.-H., Flomenberg N., Wang C., Pavlides S., Pestell R.G., Howell A., Sotgia F. (2011). Anti-estrogen resistance in breast cancer is induced by the tumor microenvironment and can be overcome by inhibiting mitochondrial function in epithelial cancer cells. Cancer Biol. Ther..

[174] Scanlan M.J., Raj B.K., Calvo B., Garin-Chesa P., Sanz-Moncasi M.P., Healey J.H., Old L.J., Rettig W.J. (1994). Molecular cloning of fibroblast activation protein alpha, a member of the serine protease family selectively expressed in stromal fibroblasts of epithelial cancers. Proc. Natl. Acad. Sci. USA.

[175] Jacob M., Chang L., Pure E. (2012). Fibroblast Activation Protein in Remodeling Tissues. Curr. Mol. Med..

[176] Pure E., Blomberg R. (2018). Pro-tumorigenic roles of fibroblast activation protein in cancer: Back to the basics. Oncogene.

[177] Jansen K., Heirbaut L., Cheng J.D., Joossens J., Ryabtsova O., Cos P., Maes L., Lambeir A.-M., De Meester I., Augustyns K. (2013). Selective Inhibitors of Fibroblast Activation Protein (FAP) with a (4-Quinolinoyl)-glycyl-2-cyanopyrrolidine Scaffold. ACS Med. Chem. Lett..

[178] Lindner T., Loktev A., Giesel F., Kratochwil C., Altmann A., Haberkorn U. (2019). Targeting of activated fibroblasts for imaging and therapy. EJNMMI Radiopharm. Chem..

[179] Kratochwil C., Flechsig P., Lindner T., Abderrahim L., Altmann A., Mier W., Adeberg S., Rathke H., Rohrich M., Winter H. (2019). (68)Ga-FAPI PET/CT: Tracer Uptake in 28 Different Kinds of Cancer. J. Nucl. Med..

[180] Giesel F.L., Kratochwil C., Lindner T., Marschalek M.M., Loktev A., Lehnert W., Debus J., Jager D., Flechsig P., Altmann A. (2019). (68)Ga-FAPI PET/CT: Biodistribution and Preliminary Dosimetry Estimate of 2 DOTA-Containing FAP-Targeting Agents in Patients with Various Cancers. J. Nucl. Med..

[181] Kumar V., Gu Y., Basu S., Berglund A., Eschrich S.A., Schabath M.B., Forster K., Aerts H.J., Dekker A., Fenstermacher D. (2012). Radiomics: The process and the challenges. Magn. Reson. Imaging.

[182] Lambin P., Rios-Velazquez E., Leijenaar R., Carvalho S., van Stiphout R.G., Granton P., Zegers C.M., Gillies R., Boellard R., Dekker A. (2012). Radiomics: Extracting more information from medical images using advanced feature analysis. Eur. J. Cancer.

[183] Aerts H.J.W.L., Velazquez E.R., Leijenaar R.T.H., Parmar C., Grossmann P., Carvalho S., Bussink J., Monshouwer R., Haibe-Kains B., Rietveld D. (2014). Decoding tumour phenotype by noninvasive imaging using a quantitative radiomics approach. Nat. Commun..

[184] Nicolasjilwan M., Hu Y., Yan C., Meerzaman D., Holder C.A., Gutman D., Jain R., Colen R., Rubin D.L., Zinn P.O. (2015). Addition of MR imaging features and genetic biomarkers strengthens glioblastoma survival prediction in TCGA patients. J. Neuroradiol..

[185] Segal E., Sirlin C.B., Ooi C., Adler A.S., Gollub J., Chen X., Chan B.K., Matcuk G.R., Barry C.T., Chang H.Y. (2007). Decoding global gene expression programs in liver cancer by noninvasive imaging. Nat. Biotechnol..

[186] Parmar C., Leijenaar R.T., Grossmann P., Rios Velazquez E., Bussink J., Rietveld D., Rietbergen M.M., Haibe-Kains B., Lambin P., Aerts H.J. (2015). Radiomic feature clusters and prognostic signatures specific for Lung and Head & Neck cancer. Sci. Rep..

[187] Jahani N., Cohen E., Hsieh M.-K., Weinstein S.P., Pantalone L., Hylton N., Newitt D., Davatzikos C., Kontos D. (2019). Prediction of Treatment Response to Neoadjuvant Chemotherapy for Breast Cancer via Early Changes in Tumor Heterogeneity Captured by DCE-MRI Registration. Sci. Rep..

[188] Jones E.F., Ray K.M., Li W., Chien A.J., Mukhtar R.A., Esserman L.J., Franc B.L., Seo Y., Pampaloni M.H., Joe B.N. (2019). Initial experience of dedicated breast PET imaging of ER+ breast cancers using [F-18]fluoroestradiol. NPJ Breast Cancer.

[189] Jones E.F., Ray K.M., Li W., Seo Y., Franc B.L., Chien A.J., Esserman L.J., Pampaloni M.H., Joe B.N., Hylton N.M. (2017). Dedicated Breast Positron Emission Tomography for the Evaluation of Early Response to Neoadjuvant Chemotherapy in Breast Cancer. Clin. Breast Cancer.

[190] McLaughlin R.L., Newitt D.C., Wilmes L.J., Jones E.F., Wisner D.J., Kornak J., Proctor E., Joe B.N., Hylton N.M. (2014). High resolution in vivo characterization of apparent diffusion coefficient at the tumor-stromal boundary of breast carcinomas: A pilot study to assess treatment response using proximity-dependent diffusion-weighted imaging. J. Magn. Reson. Imaging JMRI.

[191] Wilmes L.J., McLaughlin R.L., Newitt D.C., Singer L., Sinha S.P., Proctor E., Wisner D.J., Saritas E.U., Kornak J., Shankaranarayanan A. (2013). High-resolution diffusion-weighted imaging for monitoring breast cancer treatment response. Acad. Radiol..

[192] Cook G.J., Yip C., Siddique M., Goh V., Chicklore S., Roy A., Marsden P., Ahmad S., Landau D. (2013). Are pretreatment 18F-FDG PET tumor textural features in non-small cell lung cancer associated with response and survival after chemoradiotherapy?. J. Nucl. Med..

[193] Coroller T.P., Grossmann P., Hou Y., Rios Velazquez E., Leijenaar R.T., Hermann G., Lambin P., Haibe-Kains B., Mak R.H., Aerts H.J. (2015). CT-based radiomic signature predicts distant metastasis in lung adenocarcinoma. Radiother. Oncol..

[194] Parmar C., Grossmann P., Rietveld D., Rietbergen M.M., Lambin P., Aerts H.J.W.L. (2015). Radiomic Machine-Learning Classifiers for Prognostic Biomarkers of Head and Neck Cancer. Front. Oncol..

[195] Greenspan H., Ginneken B.v., Summers R.M. (2016). Guest Editorial Deep Learning in Medical Imaging: Overview and Future Promise of an Exciting New Technique. IEEE Trans. Med. Imaging.

[196] Shen L., Margolies L.R., Rothstein J.H., Fluder E., McBride R., Sieh W. (2019). Deep Learning to Improve Breast Cancer Detection on Screening Mammography. Sci. Rep..

[197] Lehman C.D., Yala A., Schuster T., Dontchos B., Bahl M., Swanson K., Barzilay R. (2019). Mammographic Breast Density Assessment Using Deep Learning: Clinical Implementation. Radiology.

[198] Yala A., Lehman C., Schuster T., Portnoi T., Barzilay R. (2019). A Deep Learning Mammography-based Model for Improved Breast Cancer Risk Prediction. Radiology.

[199] Wang J., Yang X., Cai H., Tan W., Jin C., Li L. (2016). Discrimination of Breast Cancer with Microcalcifications on Mammography by Deep Learning. Sci. Rep..

